# Taxonomic studies on the ant genus *Cerapachys* Smith (Hymenoptera, Formicidae) from India

**DOI:** 10.3897/zookeys.336.5719

**Published:** 2013-09-27

**Authors:** Himender Bharti, Shahid Ali Akbar

**Affiliations:** 1 Department of Zoology & Environmental Sciences, Punjabi University, Patiala, Punjab-147002 India

**Keywords:** Ants, Cerapachyinae, ergatoid queens, new species, myrmecophagy, India, Formicidae, *Cerapachys*, key, taxonomy

## Abstract

The Indianspeciesof the ant genus *Cerapachys* Smith are keyed. Twelve species are recognized of which 6 are described as new. The species are: *Cerapachys aitkenii* Forel, *Cerapachys alii* **sp. n.**, *Cerapachys anokha* **sp. n.**, *Cerapachys besucheti* Brown, *Cerapachys biroi* Forel, *Cerapachys indicus* Brown, *Cerapachys longitarsus* (Mayr), *Cerapachys nayana* **sp. n.**, *Cerapachys schoedli* **sp. n.**, *Cerapachys seema* **sp. n.**, *Cerapachys sulcinodis* Emery and *Cerapachys wighti* **sp. n.** Geographic distribution and group affinities of the new species are discussed. A revised key to the Indian species is provided. The rare ergatoid queens of *Cerapachys nayana*, *Cerapachys schoedli* and *Cerapachys seema* are reported. Formed in response to selective pressures these ergatoid queens have a significant role in dispersal strategies and contribute much to our understanding of the biology of these ants.

## Introduction

The ant genus *Cerapachys* includesmainly myrmecophagous ants which raid the nests of other ants for prey (Wilson 1959). The genus is distributed widely throughout the tropical and subtropical regions of the world, with the majority of species known from the Indo-Australian region (Brown 1975). *Cerapachys* is the largest genus in the tribe Cerapachyini and is represented by 153 species globally (Bolton 2013). The tribe was comprehensively covered by Brown (1975), with treatment of 137 described species. Other significant contributions to the genus from South-east Asia include those of Ogata (1983) who described a new species from China; Terayama et al. (1988) provided notes on the Taiwanese species, with the rediscovery of *Cerapachys sauteri* Forel, 1913 from Taiwan; Morisita et al. (1989) contributed a guide to identification of Japanese species; Radchenko (1993) described the queen of *Cerapachys sauteri* from Vietnam; Wu and Wang (1995) reviewed the Chinese species; Terayama (1996, 2009) respectively provided keys in addition to descriptions of three new species from Taiwan and Borowiec (2009) discussed the status of the spurious genus *Yunodorylus* Xu, and described three new species related to *Cerapachys sexspinus* (Xu, 2000).

*Cerapachys* in India is currently represented by 6 species (Bharti 2011). The present study reports 6 further new species; *Cerapachys alii* sp. n., *Cerapachys anokha* sp. n., *Cerapachys nayana* sp. n., *Cerapachys schoedli* sp. n., *Cerapachys seema* sp. n., and *Cerapachys wighti* sp. n. from the southwest of the country. *Cerapachys* is thus now represented by 12 Indian species, a revised key to which is provided here. The present study aims to describe and catalogue the diversity of *Cerapachys* species from India and to discuss group affinities based upon available data.

## Materials and methods

**Figure 1.**
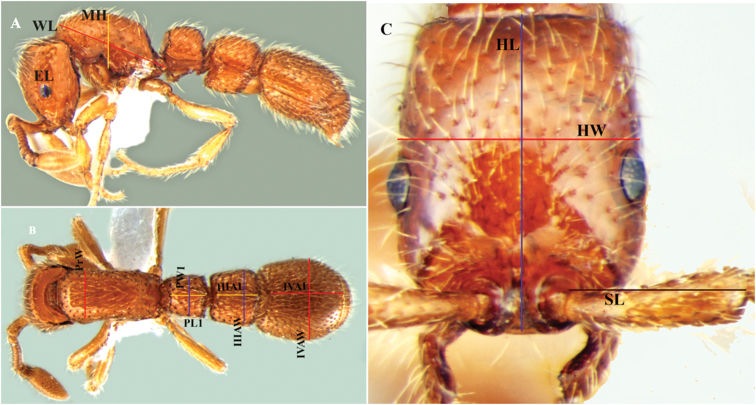
Images illustrating the measurements used. **A** body in lateral view with measuring lines for EL, MH, and WL **B** body in dorsal view with measuring lines for PrW, PL1, PW1, IIIAW, IIIAL, IVAW and IVAL **C** head in full-face view with measuring lines for HL, HW and SL.

The specimens were collected by hand and Winkler extraction. Taxonomic analysis was conducted using a Nikon SMZ 1500 stereo zoom microscope. For digital images, an MP evolution digital camera was used on the same microscope with Auto-Montage (Syncroscopy, Division of Synoptics, Ltd.) software. Later, images were cleaned as per requirement with Adobe Photoshop CS6. Description style and morphological terminology for measurements and indices follow Brown (1975) and Borowiec (2009) and include: HL = Head length; maximum length of head in dorsal view, measured in a straight line from the anterior-most point of the nasal (clypeal) flank to the midpoint of the frontovertextal margin (Fig. 1C); HW = Head width; maximum width of head in dorsal view (Fig. 1C); EL = Eye length; maximum length of eye as measured normally in oblique view of the head to show full surface of eye (Fig. 1A); SL = Scape length; maximum length of the scape excluding the basal neck and condyle (Fig. 1C); WL = Weber’s length measured from the anterior surface of the pronotum proper (excluding the collar) to the posterior extension of the propodeal lobes (Fig. 1A); MH = Mesosoma height; in the side view, maximum height measured from the lowermost point of the mesopleuron (in front of middle coxa) to the dorsal edge of the mesosoma (Fig. 1A); PrW = Pronotal width; maximum width in dorsal view (Fig. 1B); PL1 = Petiole length; maximum length of the petiole in dorsal view (Fig. 1B); PW1 = Petiole width; maximum width of the petiole in dorsal view (Fig. 1B); IIIAL = Third abdominal tergite length; Maximum length in dorsal view, measuring only the length of the Posttergite (excluding pretergite, III, helcium) (Fig. 1B); IIIAW = Third abdominal tergite width; Maximum width in dorsal view (Fig. 1B); IVAW = Fourth abdominal tergite width; Maximum width in dorsal view (Fig. 1B); IVAL = Fourth abdominal tergite length; Maximum length in dorsal view, excluding pretergite (Fig. 1B); CI = Cephalic index: HW/HL × 100; SI = Scape index: SL/HW × 100; PI = Petiolar index: PW1/PL1 × 100.

### Acronyms of depositories

BMNH Natural History Museum, London, U.K.

PUAC Punjabi University Patiala, Ant Collection at Department of Zoology and Environmental Sciences, Punjabi University, Patiala, Punjab, India.

### Key to the species of genus *Cerapachys* from India based on worker caste (modified after Brown 1975)

**Table:** 

1	Antenna 9 segmented	2
–	Antenna 11 or 12 segmented	3
2	Larger species (HW 0.46–0.49 mm). Sculpture predominantly punctuate (Fig. 2A)	*Cerapachys biroi* Forel
–	Smaller species (HW 0.37–0.39 mm). Sculpture predominantly foveate (Fig. 2B)	*Cerapachys alii* sp. n.
3	Antenna 11 segmented	*Cerapachys besucheti* Brown
–	Antenna 12 segmented	4
4	Petiole with dorsum rounding into sides; dorsolateral margins absent or vestigial (Fig. 3A)	6
–	Petiole with strong overhanging dorsolateral margins (Fig. 3B)	5
5	Head brown, trunk red or brown, petiole and postpetiole light to dark reddish, gaster brown or black. Dorsal surface of body shiny, with widely scattered, indistinct punctures, throughout the body (Fig. 4A)	*Cerapachys longitarsus* (Mayr)
–	Head, trunk, petiole and postpetiole black coloured. Dorsal surface of body shiny, with widely scattered, indistinct punctures, mostly confined to postpetiole (Fig. 4B)	*Cerapachys nayana* sp. n.
6	Dorsal surface of petiolar node with a smooth, median area (Fig. 5A)	*Cerapachys sulcinodis* Emery
–	Dorsal surface of petiolar node rounded and punctuate, without a differentiated median smooth area (Fig. 5B)	7
7	Punctures on dorsum of head relatively small, their diameter smaller than the average distance separating them (Fig. 6A)	8
–	Punctures on head dorsum large, foveiform, dense, their diameter as large, or larger than, the average distance separating them, and in some cases these are contiguous (Fig. 6B)	10
8	Declivity of propodeum with distinct cariniform margins, continuous across the dorsum; petiolar dorsum flat; colour other than black (Fig. 7A)	9
–	Declivity of propodeum without distinct cariniform margins, petiolar dorsum strongly convex; colour black (Fig. 7B)	*Cerapachys anokha* sp. n.
9	Shiny species; body sculpture reduced; eyes breaking the lateral margins of head; colour varies from light orange to dark red (Fig. 8A)	*Cerapachys schoedli* sp. n.
–	Dull coloured species; body sculpture prominent; eyes not breaking the lateral margins of head; colour brown to dark brown (Fig. 8B)	*Cerapachys seema* sp. n.
10	Eyes reduced (EL<0.1mm; Fig. 9A)	*Cerapachys wighti* sp. n.
–	Eyes large (EL>0.2mm; Fig. 9B)	11
11	Head reddish brown or red; trunk and both nodes red; gaster black or dark brown; dorsal surface of mesosoma densely and finely sculptured; foveate or rugo-reticulate (Fig. 10A)	*Cerapachys aitkenii* Forel
–	Body unicolorous, lighter brownish red; dorsal surface of mesosoma mostly smooth with few scattered punctures along sides (Fig. 10B)	*Cerapachys indicus* Brown

**Figure 2.**
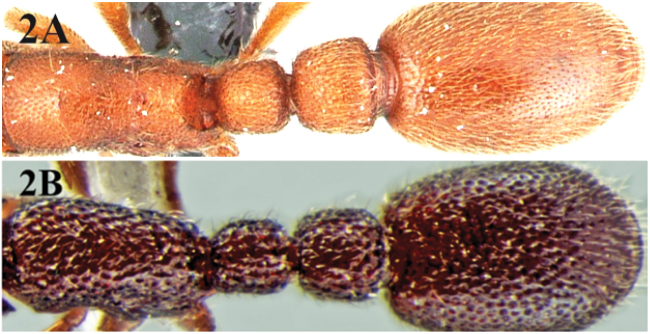
**A** dorsal surface of *Cerapachys biroi* with conspicuous punctate sculpture **B** dorsal surface of *Cerapachys alii* with foveate sculpture.

**Figure 3.**
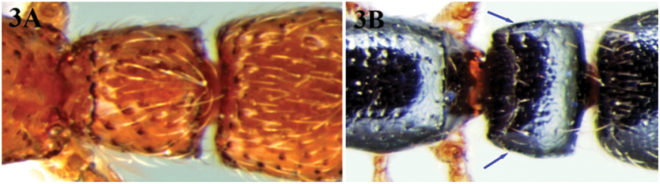
**A** petiole of *Cerapachys schoedli* with dorsum rounding into sides without overhanging dorsolateral margins **B** petioleof *Cerapachys nayana* with strong overhanging dorsolateral margins.

**Figure 4.**
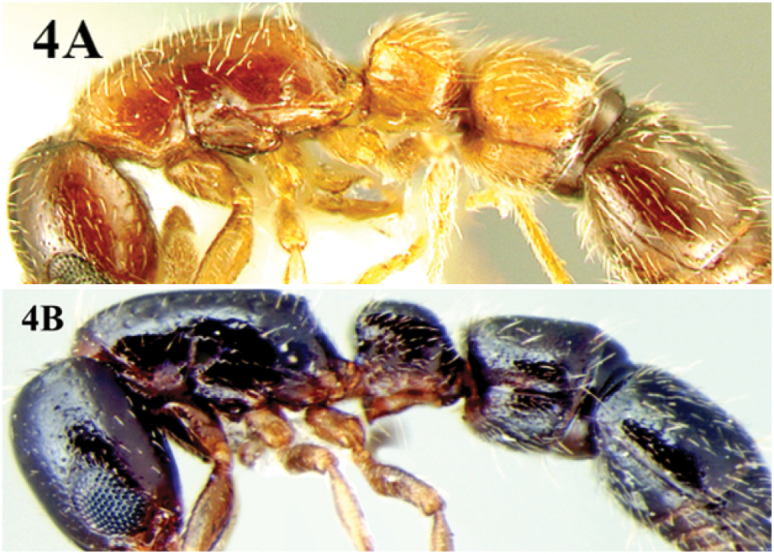
**A**. body of *Cerapachys longitarsus* in lateral view illustrating the characteristic bicolouration **B** uniformly coloured body of *Cerapachys nayana* in lateral view.

**Figure 5.**
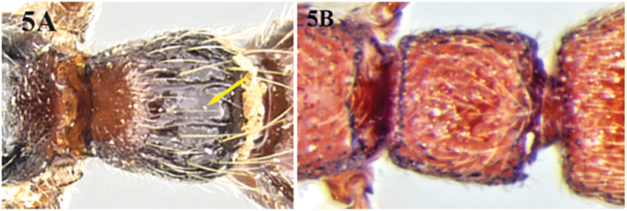
**A** petiolar node of *Cerapachys sulcinodis* with smooth median area **B** petiolar node of *Cerapachys wighti* without any median smooth area.

**Figure 6.**
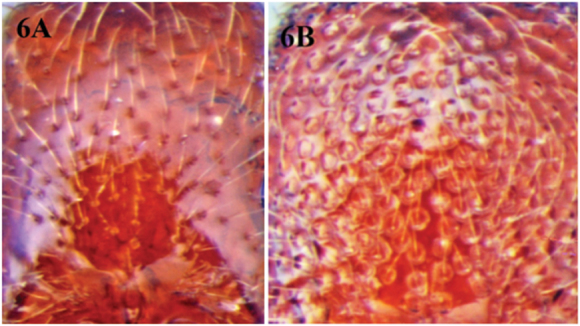
**A** cephalic dorsum of *Cerapachys schoedli* illustrating small punctures with diameter less than the average distance separating them**B** cephalic dorsum of *Cerapachys wighti* showing large punctures with diameter greater than space separating them.

**Figure 7.**
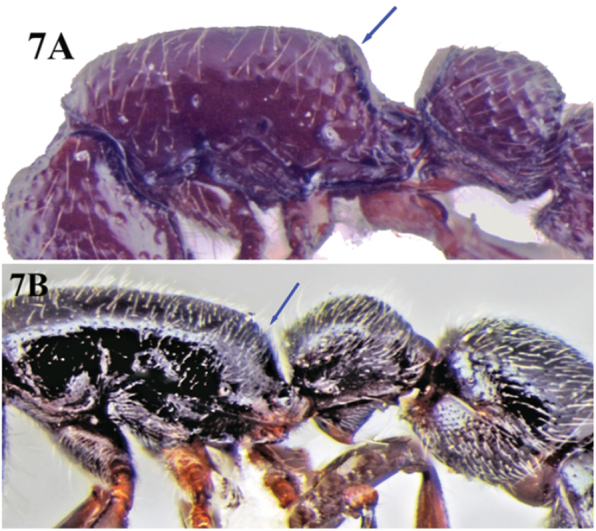
**A** body profile of *Cerapachys indicus* in lateral view showing declivity of propodeum with distinct cariniform margins **B** body profile of *Cerapachys anokha* in lateral view with propodeal declivity lacking distinct cariniform margins.

**Figure 8.**
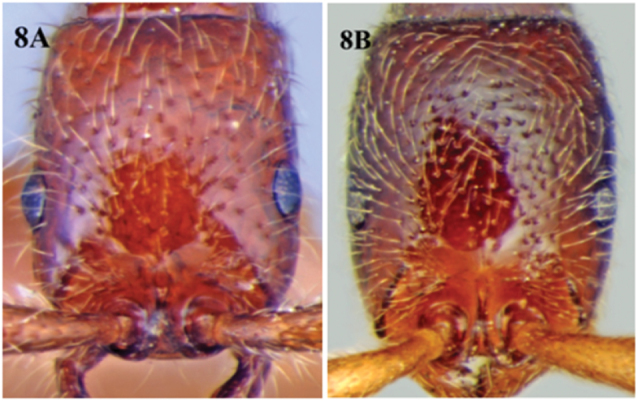
**A** shining cephalic dorsum of *Cerapachys schoedli* with reduced sculpture and eyes not breaking the lateral margins of head **B** dull cephalic dorsum of *Cerapachys seema* with prominent sculpture and eyes breaking the lateral margins of head.

**Figure 9.**
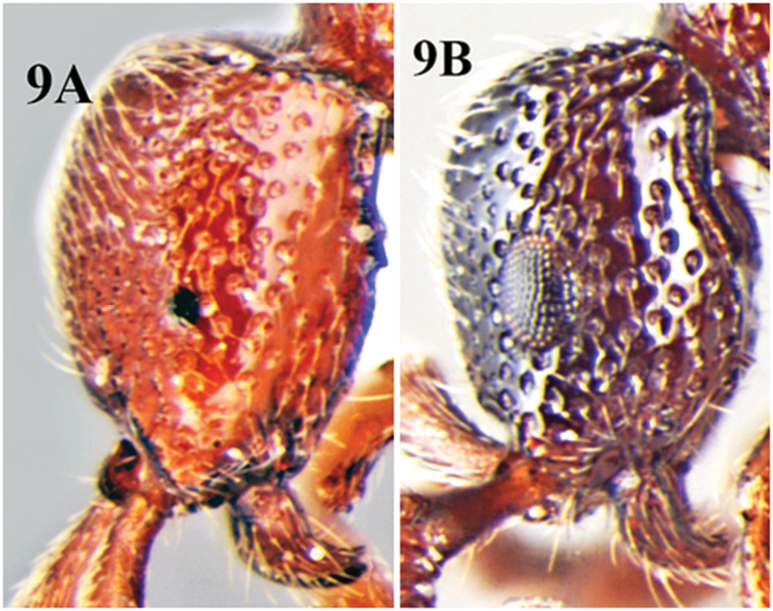
**A** head of *Cerapachys wighti* in lateral view illustrating smaller eyes **B** head of *Cerapachys aitkenii* in lateral view illustrating larger eyes.

**Figures 10.**
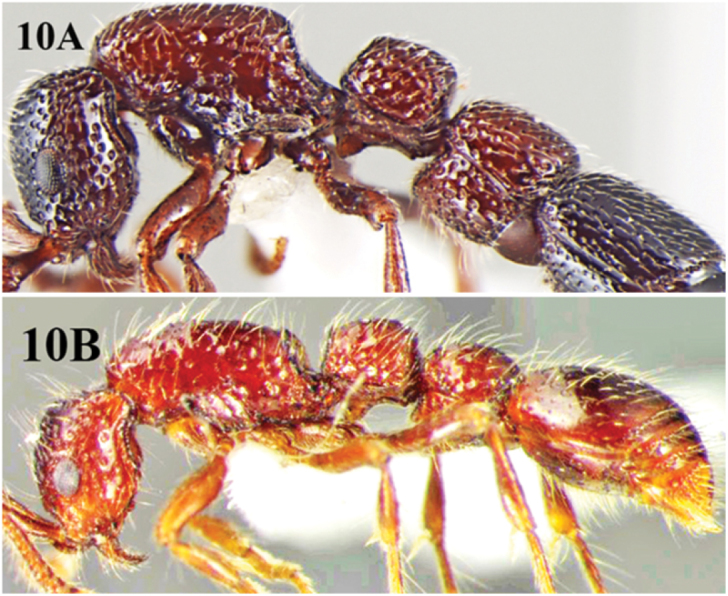
**A** body profile of *Cerapachys aitkenii* in lateral view illustrating the characteristic bicolouration and prominent foveate sculpture **B** body profile of *Cerapachys indicus* in lateral view illustrating unicolorous body colouration and with reduced punctuate sculpture.

## Results

### Descriptions of new species

#### Cerapachys alii sp. n.

http://zoobank.org/52540038-860B-4EA4-BA1E-40217FF6D555

http://species-id.net/wiki/Cerapachys_alii

**Figures 11–13.**
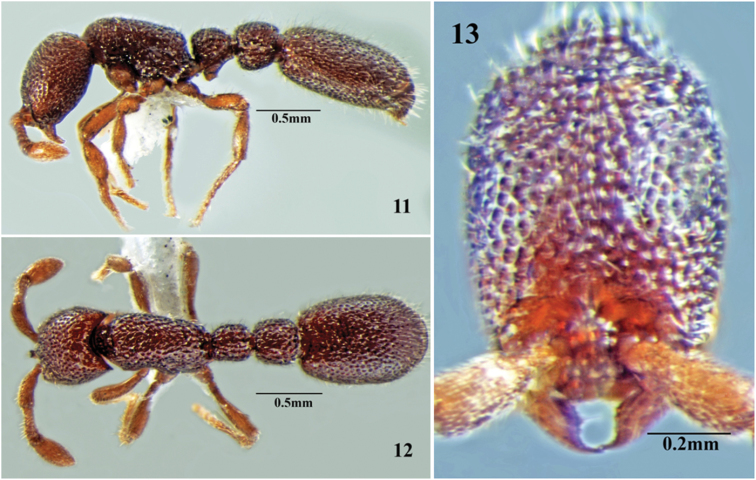
*Cerapachys alii* sp. n. **11** body in profile **12** body in dorsal view **13** head in full-face view.

Figures 2B , 11 , 12 , 13 , Table 1

##### Type material.

Holotype and 6 paratypes (worker): India, Kerala, Salim Ali Bird Sanctuary, 10°45'N, 76°44'E, 118m a.s.l., 10.x.2011, Winkler method (coll. Shahid A. Akbar); Holotype in PUAC and paratype in BMNH.

##### Worker description.

Measurements (holotype in brackets): HL 0.46-0.51 (0.48); HW 0.37-0.39 (0.38); WL 0.47-0.49 (0.49); MH 0.28-0.31 (0.31); PrW 0.25-0.29 (0.25); PL1 0.16-0.20 (0.17); PW1 0.16-0.19 (0.18); IIIAL 0.20-0.22 (0.22); IIIAW 0.22-0.27 (0.23); SL 0.21-0.22 (0.22); IVAL 0.43-0.49 (0.49); IVAW 0.39-0.41 (0.41). Indices: CI 76-80 (79); SI 56-57 (57); PI 95-105 (105) (n=5).

Head. Rectangular, longer than broad, sides converge anteriorly; vertexal margin concave, posterior lateral corners rounded. Parafrontal ridges prominent, raised. Eyes absent. Mandibles dentate; narrow, with strongly incurved apical tooth; anterior clypeal margin entire and projects forward as a low rounded transparent lobe or apron. Lateroclypeal teeth reduced. Antennae 9 segmented; scapes short, clavate, each falling short of posterior margin of head by 1/3^rd^ of its length.

Mesosoma. Stout, wider anteriorly; dorsal surface slightly convex, almost flat, the dorsal surface gently rounded along sides without any distinct margin. Declivous face of propodeum with cariniform margins across the top and along lateral margins.

Metasoma. Petiole as long as broad, without overhanging dorsolateral margins. Anterior face transverse and posterior face shallowly convex. Subpetiolar process prominent, acute, posteriorly directed; no fenestra present. Postpetiole slightly longer than broad, lateral angles uniformly rounded. Gaster elongate; base of cinctus of first gastral tergite with cross ribs; sting exerted.

Sculpture. Mandibles punctured. Head strongly foveate. Mesosoma, petiole and postpetiole with similar prominent foveate sculpture.

Vestiture. Body with reduced white pilosity; moderate, decumbent or subdecumbent hairs distributed evenly throughout. Apical funicular segments and legs with small standing hairs.

Colour. Dark red with mandibles, antennae and legs castaneous.

##### Etymology.

The species is named in honor of Dr. Salim Ali, renowned Indian Ornithologist.

##### Differential diagnosis.

With its 9 segmented antennae *Cerapachys alii* can be easily separated from other species known from India. Only eight other known species of *Cerapachys* are reported to have 9 segmented antennae. These eight species are placed in the *typhlus* group and include; *Cerapachys biroi* Forel, 1907; *Cerapachys cryptus* Mann, 1921; *Cerapachys edentatus* Forel, 1900; *Cerapachys fuscior* Mann, 1921; *Cerapachys papuanus* Emery, 1897; *Cerapachys pawa* Mann, 1919; *Cerapachys pusillus* Emery, 1897 and *Cerapachys typhlus* Roger, 1861. The new species can be easily separated from all of them. *Cerapachys cryptus* and *Cerapachys fuscior* are larger species (HW > 0.70 mm) while *Cerapachys alii* is a smaller species (HW< 0.40 mm). *Cerapachys typhlus* has the postpetiole more than half as long as the succeeding gastric segment while in *Cerapachys alii* it is less than half as long as the succeeding gastric segment. *Cerapachys papuanus*, *Cerapachys pawa* and *Cerapachys pusillus* have the anterolateral shoulders of the first gastric segment abruptly rounded, accentuating the medium concavity that receives the postpetiole while in *Cerapachys alii* theanterolateral shoulders of the first gastric segments as seen from above broadly rounded and gradually widening caudad. *Cerapachys biroi* and *Cerapachys edentatus* predominantly have punctuate body sculpture while *Cerapachys alii* has predominantly foveate body sculpture. *Cerapachys alii* can also be confused with *Cerapachys fragosus* Roger, 1862 and *Cerapachys coecus* Mayr, 1897 which has similar prominent foveate body sculpture, however these two species are characterized by 11 segmented antennae while as *Cerapachys alii* has 9 segmented antennae.

**Ecology.** This subterranean species seems to be of rare occurrence as it was encountered only once during the extensive surveys in the region. The specimens were collected from a leaf litter sample taken from Salim Ali bird Sanctuary. A low-land evergreen forest area, located between the branches of Periyar river. The region is considered as the richest bird habitat on peninsular India.

**Table 1.** Distribution of *Cerapachys* species. EQ=ergatoid queens; Q=queens; M=male; W=worker; + indicates reported and – indicates not reported.

**N**	**Species**	**Distribution**	**EQ**	**Q**	**M**	**W**
1	*Cerapachys alii* sp. n.	India, Kerala	-	-	-	+
2	*Cerapachys anokha* sp. n.	India, Kerala	-	-	-	+
3	*Cerapachys aitkenii* Forel, 1900	India, Kerala	-	-		+
4	*Cerapachys besucheti* Brown, 1975	India, Tamil Nadu	+	-	-	+
5	*Cerapachys biroi* Forel, 1907	Madagascar, Philippines, Puerto Rico, China, Nepal, India, Comoros, Seychelles, Mayotte, Japan, Viet Nam, Guam, Samoa, Marshall Islands, Northern Mariana Islands and Hawaii	+	+	+	+
6	*Cerapachys longitarsus* (Mayr, 1879)	Philippines, New Guinea, Israel, India, Bangladesh, Thailand, Saudi Arabia, Egypt, United Arab Emirates	+	+	+	+
7	*Cerapachys nayana* sp. n.	India, Karnataka, Kerala	+	-	-	+
8	*Cerapachys indicus* Brown, 1975	India, Kerala	-	+	-	+
9	*Cerapachys schoedli* sp. n.	India, Kerala	-	-	-	+
10	*Cerapachys seema* sp. n.	India, Kerala	+	+	-	+
11	*Cerapachys sulcinodis* Emery, 1889	Philippines, Indonesia China, India, Viet Nam and Thailand	+	+	+	+
12	*Cerapachys wighti* sp. n.	India, Kerala	-	-	-	+

#### Cerapachys anokha sp. n.

http://zoobank.org/2A8BE8D2-BED1-4DAC-A73F-E9AC2189FE23

http://species-id.net/wiki/Cerapachys_anokha

**Figures 14–16.**
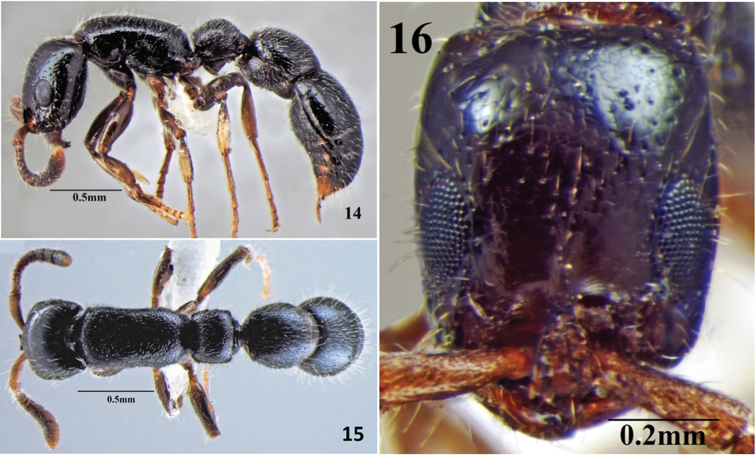
*Cerapachys anokha* sp. n. **14** body in profile **15** body in dorsal view. **16**. head in full-face view.

Figures 7B , 14 , 15 , 16 , Table 1

##### Type material.

Holotype and 3 paratypes (worker): India: Kerala, Periyar tiger reserve, Thanikkudy, 9°.30'N, 77°.16'E, 1003m a.s.l., 15.x.2011, hand picking method (coll. Shahid A. Akbar). Holotype in PUAC and paratype in BMNH.

##### Worker description.

Measurements (holotype in brackets): HL 0.69-0.73 (0.72); HW 0.60-0.63 (0.63); EL 0.20-0.22 (0.22); WL 0.72-0.80 (0.80); MH 0.33-0.38 (0.38); PrW 0.42-0.47 (0.47); PL1 0.29-0.33 (0.33); PW1 0.38-0.41 (0.41); IIIAL 0.38-0.45 (0.41); IIIAW 0.44-0.55 (0.52); SL 0.29-0.32 (0.32); IVAL 0.85-0.92 (0.92); IVAW 0.57-0.64 (0.64). Indices: CI 86-87 (87); SI 48-51 (51); PI 124-131 (124) (n=4).

Head. Rectangular, longer than broad, widest at about mid-length; sides parallel; vertexal margin slightly concave, posterior lateral corners are weakly acute to rounded. Parafrontal ridges present. Eyes prominent, placed below midline of head. Mandible triangular with acute apices and sharp concave, edentate, masticatory margins; anterior clypeal margin with small apron shaped transparent structure. Lateroclypeal teeth small, blunt and projecting slightly inwards. Antennae 12 segmented; scape short and clavate, reaching up to 1/3^rd^ of posterior margin of head.

Mesosoma. Stout, rectangular in dorsal view; dorsal surface convex, the dorsal surface gently rounded along sides without any distinct margin. Declivous face of propodeum lacking cariniform margins across the top and along sides.

Metasoma. Petiole highly convex, broader than long, with traces of reduced dorsolateral margins, anterior and posterior faces continuous with dorsum. Subpetiolar process prominent, wedge like, with apex directed backward; no fenestra present. Postpetiole wider than long, uniformly rounded. Gaster elongate; base of cinctus of first gastral tergite with cross ribs; sting exerted.

Sculpture. Mandibles smooth and shining. Head with few small punctures. Sculpture on dorsal surface of mesosoma, petiole and postpetiole consist of very small, uniform punctures, distributed throughout the surface. Gaster mostly smooth, with few scattered punctures. Cinctus of 1^st^ gastral, with few cross ribs.

Vestiture. Body covered with decumbent or subdecumbent hairs. Longer hairs are also present on postpetiole and gaster. Head also consists of few long hairs; apical funicular segments and legs with standing hairs.

Colour. Black with mandibles, antennae and legs castaneous.

##### Etymology.

The species epithet is Hindi for “unique”, in reference to its unique nature of propodeal declivity.

##### Differential diagnosis.

This species is unique in having the declivous face of the propodeum lacking cariniform margins across the top and along the sides, features unique in described workers of the Cerapachyinae. The new species show interesting variation in the form of the petiole. The petiolar node has the inferior as well as the superior posterolateral angles produced, but not sharply angular. The sides of the petiole could not be considered either immarginate (*Cerapachys* lineage) or marginate (*Phyracaces* lineage). This makes the placement of this species somewhat transitional between the two lineages and easily distinguishes it from other reported species of the genus. When using Brown’s (1975) key *Cerapachys anokha* comes close to *singaporensis* Viehmeyer, 1916. The two species however can be easily separated. *Cerapachys singaporensis* has the body arrayed with long pale hairs; and copiously pubescent, and the dorsal sides of the petiole strongly marginate, while *Cerapachys anokha* has only decumbent or subdecumbent body hairs, little pubescence and the dorsolateral sides of petiole are not marginate. *Cerapachys anokha* could also be confused with *Cerapachys nayana* which has similar habitat preferences and body colouration; however the two species can be easily separated: *Cerapachys nayana* has larger eyes (EL 0.24–0.27 mm), the declivous face of the propodeum has cariniform margins across the top, and the petiole has marginate dorsolateral sides; while *Cerapachys anokha* has smaller eyes (EL 0.20–0.22 mm), the declivous face of its propodeum lacks cariniform margins across the top, and the petiole is without marginate dorsolateral sides.

##### Ecology.

This species seems to be infrequent. It is from the Western Ghats. Four specimens were collected by handpicking from the Thanikkudy region of the Periyar tiger reserve. Which is a primary, undisturbed tropical moist evergreen forest. The area is situated at 1003 meters elevation. It is a shady place with little sunlight penetration.

#### Cerapachys nayana sp. n.

http://zoobank.org/46F8FF78-D753-4BEF-8FCB-7A83D4149E8D

http://species-id.net/wiki/Cerapachys_nayana

Figures 3B , 4B , 17 , 18 , 19 , 20 , 21 , 22 , Table 1

##### Type material.

Holotype worker: India: Kerala, Silent valley national park, 11°5'N, 76°26'E, 897m a.s.l., 25.ix.2011, hand picking. Paratypes: 2 workers and 1 ergatoid queen with same data as holotype; 6 workers and 2 ergatoid queens, India, Karnataka, Gundlupet 11°8'N, 76°68'E, 800m a.s.l., 27.ix.2010, hand picking; 2 workers and 1 ergatoid queen, India, Kerala, Periyar tiger reserve, 9°46'N, 77°14'E, 1005m a.s.l., 10.x.2011, hand picking (coll. Shahid A. Akbar). Holotype in PUAC and paratype in BMNH.

##### Worker description.

Measurements (holotype in brackets): HL 0.55-0.66 (0.66); HW 0.48-0.51 (0.51); EL 0.24-0.27 (0.25); WL 0.60-0.66 (0.66); MH 0.34-0.37 (0.34); PrW 0.33-0.38 (0.34); PL1 0.20-0.23 (0.23); PW1 0.25-0.29 (0.29); IIIAL 0.30-0.44 (0.44); IIIAW 0.37-0.47 (0.37); SL 0.21-0.27 (0.27); IVAL 0.58-0.61 (0.61); IVAW 0.47-0.52 (0.52). Indices: CI 77-87 (77); SI 44-53 (53); PI 125-126 (126) (n=10).

Head. Rectangular, longer than broad, widest at about its midlength; sides parallel, vertexal margin transverse to shallowly concave, posterior lateral corners weakly acute. Parafrontal ridges present but not raised, very low. Eyes large prominent. Mandibles subtriangular; masticatory margin without a row of small denticles. Lateroclypeal teeth small and reduced. Antennae 12 segmented; scapes short, reaching up to 4/5^th^ of posterior margin of head.

Mesosoma. Moderately stout, rectangular in dorsal view; dorsal surface flattened, bordered laterally by a distinct angle, but no margin. Declivous face of propodeum with cariniform margins across the top and along the lateral margins.

Metasoma. Petiole broader than long, with strong overhanging dorsolateral margins. Anterior face concave while posterior face is transverse. Subpetiolar process small with stout acute apex, directed forward, located beneath anterior 1/3rd of the petiole; no fenestra present. Postpetiole sub trapezoidal, wider behind with the posterolateral angles uniformly rounded. Gaster elongate; base of cinctus of first gastral tergite with cross ribs; sting exerted.

Sculpture. Mandibles smooth and shining. Head with small punctures, spaced wider than their diameter, dorsum of head also with faint rugae in between the punctures. Similar sculpture on dorsal surface of mesosoma and petiole. Small continuous punctures produce a matt like appearance on the dorsum of the postpetiole. Gaster with similar matt-like appearance but less prominent. Cinctus of 1^st^ gastral segment smooth and shining.

Vestiture. Body covered with moderate decumbent or subdecumbent hairs most prominent on postpetiole and gaster, head devoid of such hairs, only a few along sides; apical funicular segments with standing hairs.

Colour. Black with mandibles, antennae and legs castaneous.

**Figures 17–19.**
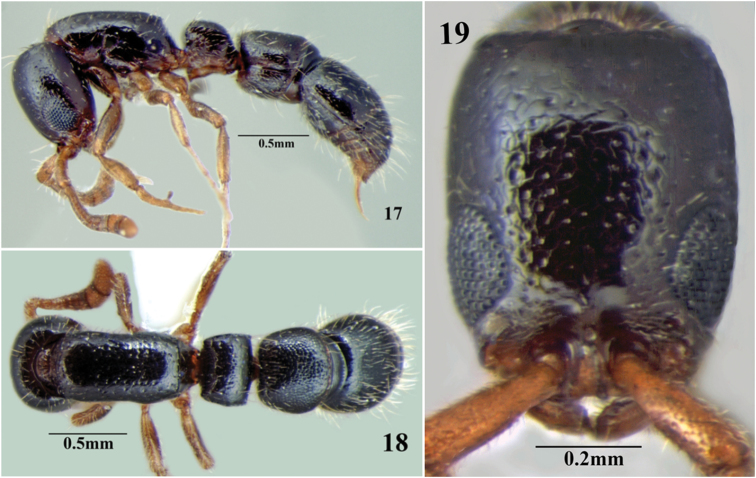
*Cerapachys nayana* sp. n. **17** body in profile **18** body in dorsal view **19** head in full-face view.

**Figures 20–22.**
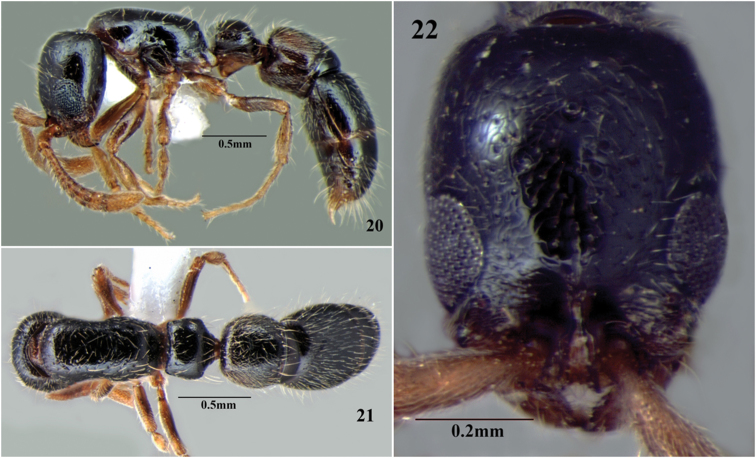
*Cerapachys nayana* sp. n. ergatoid queen. **20** body in profile **21** body in dorsal view **22** head in full-face view.

##### Ergatoid queen measurements.

HL 0.66-0.77; HW 0.55-0.60; EL 0.22-0.24; WL 0.79-0.82; MH 0.36-0.41; PrW 0.44-0.47; PL1 0.24-0.27; PW1 0.44-0.47; IIIAL 0.44-0.47; IIIAW 0.51-0.55; SL 0.18-0.22; IVAL 0.58-0.63; IVAW; 0.60-0.66. Indices: CI 77-83; SI 33-37; PI 174-183 (n=3).

Like the workers of the same colony, but larger, with thicker body, especially mesosoma and gaster. Ocelli present on vertex, prominent. The pilosity is much more prominent compared to workers. Distinction between ergatoid queens and worker is vague, with size variation of workers very high.

##### Variations.

There is a considerable amount of size variation between individual specimens, the smaller workers are lighter in body colouration compared with larger specimens; the body sculpture and pilosity also differs between individuals.

##### Etymology.

The species epithet is Sanskrit for “eyes”, in reference to the large eye size of the species.

##### Differential diagnosis.

With themarginate dorsolateral sides of its petiole, this species is easily distinguished from other Indian species of its genus. The new species sharesmost characters with *Cerapachys anokha* from which it is separated by the combination of characters given in the diagnosis of the latter species. *Cerapachys nayana* is compared with *Cerapachys longitarsus* which also has marginate dorsolateral sides to the petiole; however, the two species can be easily separated. *Cerapachys longitarsus* has characteristic bicolouration, with the head brown, trunk red or brown, petiole and postpetiole light to dark reddish and the gaster brown or black, while *Cerapachys nayana* is uniformly black coloured with mandibles, antennae and legs castaneous. The new species also resembles the Philippines, *Cerapachys luzuriagae* (Wheeler & Chapman, 1925) but can be easily separated from it. *Cerapachys nayana* is coloured black with the petiole broader than long, and with concave anterior and transverse posterior faces, the postpetiole with dense punctures and without dentition; while *Cerapachys luzuriagae* is reddish brown with the petiole as long as broad, with convex anterior and truncate posterior faces; postpetiole without dense punctures, and mandibles with prominent dentition.

##### Ecology.

This species is widely distributed in the Western Ghats. It was collected from non-forested and forest habitats from small bushes, and foraging over dry soil surfaces.

#### Cerapachys schoedli sp. n.

http://zoobank.org/38E7EC5E-E9F2-4FCC-B467-10E9208AA155

http://species-id.net/wiki/Cerapachys_schoedli

Figures 1 , 3A , 6A , 8A , 23 , 24 , 25 , 26 , 27 , 28 , Table 1

##### Type material.

Holotype worker: India. Kerala, Silent valley national park, 11°5'N, 76°26'E, 897m a.s.l., 25.ix.2011, Winkler. Paratypes: 13 workers and 3 ergatoid queens, same data as holotype; 2 workers, India, Kerala, Salim Ali Bird Sanctuary, 10°45'N, 76°44'E, 118m a.s.l., 6.xi.2011, Winkler; 10 workers, India, Kerala, Periyar tiger reserve, Manalar, 9°35'N, 77°18'E, 1630m a.s.l., 27.x.2011, hand picking (coll. Shahid A. Akbar). Holotype in PUAC and paratype in BMNH.

##### Worker description.

Measurements (holotype in brackets): HL 0.64-0.68 (0.68); HW 0.44-0.46 (0.46); EL 0.07-0.11 (0.11); WL 0.62-0.69 (0.69); MH 0.41-0.48 (0.48); PrW 0.31-0.35 (0.35); PL1 0.24-0.27 (0.27); PW1 0.28-0.31 (0.31); IIIAL 0.28-0.32 (0.32); IIIAW 0.39-0.41 (0.41); SL 0.31-0.33 (0.33); IVAL 0.70-0.74 (0.74); IVAW 0.58-0.60 (0.60). Indices: CI 67-69 (67); SI 70-72 (72); PI 114-116 (114) (n=11).

Head, rectangular, longer than broad; sides parallel; vertexal border transverse. Posterior lateral corners acute. Parafrontal ridges present, raised. Eyes medium sized, almost circular. Mandibles subtriangular; masticatory margins without a row of small denticles. Lateroclypeal teeth small. Antennae 12 segmented; scapes short, each falling short of posterior margin of head by 1/3^rd^ of its length.

Mesosoma. stout, humped in profile view; dorsal surface convex, continuous with sides, no lateral margins. Declivous face of propodeum with cariniform margins across the top and along the lateral margins.

Metasoma. Petiole broader than long, and gently rounded towards the sides. Anterior and posterior faces transverse. Subpetiolar process stout, fenestra present. Postpetiole almost rectangular, wider behind, with the posterolateral angles not tuberculate but uniformly rounded. Gaster elongate; base of cinctus of first gastral tergite with cross ribs; sting exerted.

Sculpture. Mandibles with small punctures. Head smooth and shining with some punctures. Mesosoma mostly smooth and shining with some punctures along the sides. Petiole, postpetiole and gaster with continuous punctures.

Vestiture. Body covered with moderate, decumbent or subdecumbent hairs, most prominent on gaster; apical funicular segments and legs also with standing hairs.

Colour. Bright yellowish orange to dark red.

**Figures 23–25.**
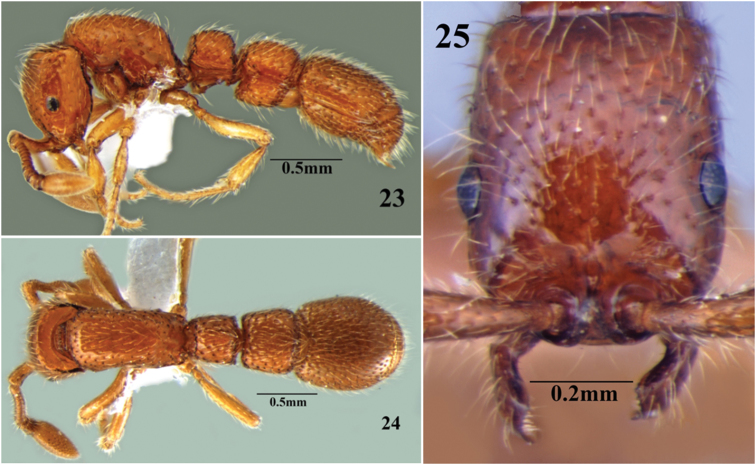
*Cerapachys schoedli* sp. n. **23** body in profile **24** body in dorsal view **25** head in full-face view.

**Figures 26–28.**
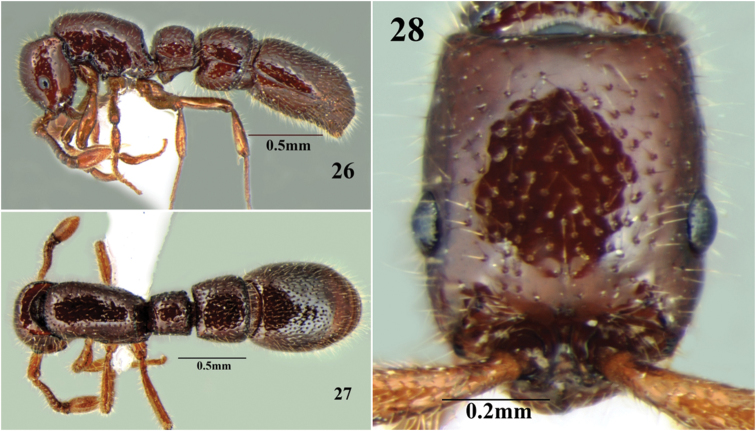
*Cerapachys schoedli* sp. n. ergatoid queen **26** body in profile **27** body in dorsal view **28** head in full-face view.

##### Ergatoid queen measurements.

HL 0.80-0.84; HW 0.59-0.63; EL 0.14-0.16; WL 0.88-0.92; MH 0.51-0.55; PrW 0.49-0.53; SL 0.41-0.43; PL1 0.29-0.31; PW1 0.38-0.41; IIIAL 0.44-0.50; IIIAW 0.57-0.59; IVAL 0.88-0.90; IVAW 0.86-0.88. Indices: CI 73-75; SI 68-69; PI 131-132 (n=3).

Like the workers of the same colony, but larger, with more stout body, especially the mesosoma and gaster. Ocelli absent on vertex. Distinction between ergatoid queens and worker is vague, with size variation of workers very high.

##### Etymology.

The species is named in the honor of the late Dr. Stefan Schödl.

##### Differential diagnosis.

This species is aberrant in many characters, with the cephalic dorsum bearing small punctures with average diameter lesser than the average distance separating them, a highly shinning body and reduced body sculpture, which separates it from other reported Indian species. *Cerapachys schoedli* sharesmost characters with *Cerapachys seema*, from which it can be easily distinguished by the combination of characters given in the diagnosis of the latter species. The new species can also be compared with *Cerapachys luteoviger* Brown, 1975 which also has small punctures on the cephalic dorsum, with diameter lesser than the average distance separating them. However, the peculiar petiolar node (with anterodorsal border concavely emarginate) and rounded head shape of *Cerapachys luteoviger* easily separates it from *Cerapachys schoedli*, which has the petiolar node broader than long and the posterior lateral corners of the head acute.

##### Ecology.

This species seems to be common in the Western Ghats; it was collected in non-forest as well as forest habitats in leaf litter and on dry soil surfaces.

#### Cerapachys seema sp. n.

http://zoobank.org/AE131489-5514-422C-BE6E-5BDBCCA2F111

http://species-id.net/wiki/Cerapachys_seema

Figures 8B , 29 , 30 , 31 , 32 , 33 , 34 , 35 , 36 , 37 , 41 , Table 1

##### Type material.

Holotype worker: India. Kerala, Periyar tiger reserve, Manalar, 9°35'N, 77°18'E, 1630m a.s.l., 24.x.2011, hand picking. Paratypes: 4 workers, 3 ergatoid queens and 1 gyne, same data as holotype (coll. Shahid A. Akbar). Holotype in PUAC and paratype in BMNH.

##### Worker description.

Measurements (holotype in brackets): HL 0.72-0.74 (0.74); HW 0.52-0.56 (0.56); EL 0.07-0.19 (0.19); WL 0.77-0.82 (0.77); MH 0.38-0.45 (0.45); PrW 0.37-0.40 (0.38); PL1 0.23-0.29 (0.26); PW1 0.36-0.41 (0.41); IIIAL 0.41-0.46 (0.41); IIIAW 0.51-0.54 (0.51); SL 0.33-0.41 (0.41); IVAL 0.70-0.74 (0.74); IVAW 0.62-0.69 (0.63). Indices: CI 72-75 (75); SI 63-73 (73); PI 141-157 (157) (n=9).

Head, longer than broad; sides converging posteriorly; vertexal margin transverse. Posterior lateral corners weakly acute. Parafrontal ridges prominent, raised. Eyes small. Mandibles subtriangular; masticatory margins deflexed and downcurved, with a row of small denticles. Lateroclypeal teeth prominent. Antennae 12 segmented; scapes clavate, reaching up to 2/3^rd^ the distance to the posterior margin of head.

Mesosoma moderately stout, rectangular in dorsal view; dorsal surface flattened and gently rounded towards the sides. Declivous face of propodeum with cariniform margins across the top and along its lateral margins.

Metasoma, Petiole broader than long, lacking dorsolateral margins. Anterior and posterior faces transverse. Subpetiolar process well developed, located below the anterior 1/3^rd^ of the petiole; fenestra present. Postpetiole broader than long with posterolateral angles uniformly rounded. Gaster elongate; base of cinctus of first gastral tergite with cross ribs; sting exerted.

Sculpture. Mandibles smooth with few punctures. Head with prominent punctures, spaced more widely than their diameter. Similar sculpture on dorsal surface of mesosoma. Petiole and postpetiole with larger punctures, forming a rugae-like surface on the dorsum. Gaster with similar sculpture to mesosoma. Cinctus of 1^st^ gastral segment with prominent transverse ribs.

Vestiture. Whole body covered with dense decumbent or subdecumbent yellowish hairs, sides of head and mesosoma with fewer hairs; apical funicular segments and legs with standing hairs.

Colour. Dark brownish black with mandibles, antennae and legs castaneous.

**Figures 29–31.**
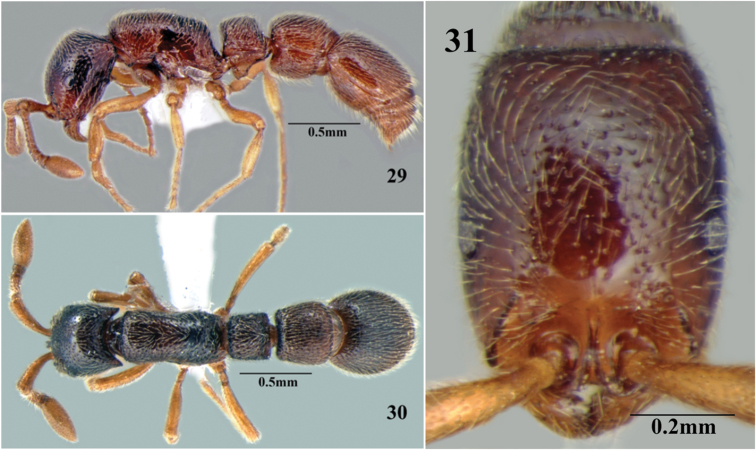
*Cerapachys seema* sp. n. **29** body in profile **30** body in dorsal view **31** head in full-face view.

**Figures 32–34.**
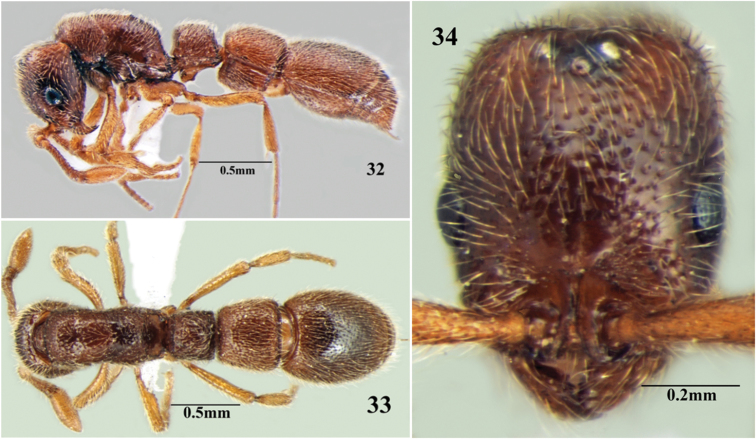
*Cerapachys seema* sp. n. ergatoid queen. **32** body in profile **33** body in dorsal view **34** head in full-face view.

**Figures 35–37.**
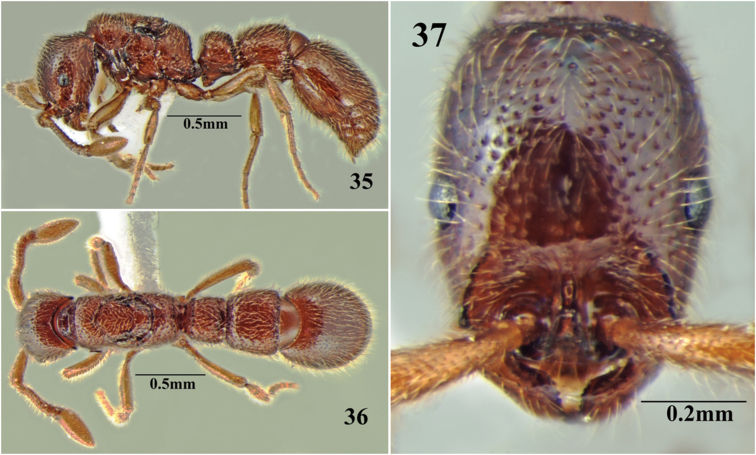
*Cerapachys seema* sp. n. queen. **35** body in profile **36** body in dorsal view **37** head in full-face view.

##### Ergatoid queen measurements.

HL 0.73-0.82; HW 0.55-0.60; EL 0.14-0.18; WL 0.81-0.86; PL1 0.27-0.29; MH 0.36-0.41; PrW 0.41-0.44; PW1 0.36-0.39; IIIAL 0.44-0.50; IIIAW 0.51-0.53; SL 0.39-0.42; IVAL 0.75-0.76; IVAW 0.72-0.77. Indices: CI 73-75; SI 70-71; PI 133-134 (n=3).

Like the workers of the same colony, but larger, with a more stout body, especially the mesosoma and gaster. Ocelli present on vertex, prominent. The pilosity is much more prominent when compared with the workers. Distinction between ergatoid queens and workers is vague with size variation of workers very high.

##### Gyne measurements.

HL 0.77; HW 0.55; EL 0.08; WL 0.93; MH 0.44; PrW 0.41; PL1 0.27; PW1 0.36; IIIAL 0.38; IIIAW 0.49; SL 0.38; IVAL 0.76; IVAW 0.73. Indices: CI 71; SI 69; PI 133 (n=1).

Resembles the worker, with modifications expected for caste and the following differences; three prominent ocelli present on vertex, thicker body with heavy pilosity and prominent sculpture.

##### Etymology.

The species epithet is Hindi for border, in reference to its type locality, Manalar, a place which marks border between Kerala and Tamil Nadu.

##### Differential diagnosis.

Thespecies is characterized by the punctures on the dorsum of the head being relatively small, separated, with their diameter smaller than the average distance separating them. The new species sharesmost characters with *Cerapachys schoedli*. However the two species can be easily separated. *Cerapachys seema* has dull body colouration, sculpture much more prominent and course, pilosity denser, eyes not breaking the lateral margins of head and head almost oval, with anterior and posterior sections of the sides converging, while *Cerapachys schoedli* is brightly coloured, its sculpture and pilosity are reduced, its eyes break the lateral margins of the head and the head is rectangular with parallel sides.

##### Ecology.

Manalar, part of Periyar tiger reserve, the type locality of this species is a fascinating green hill station (with plenty of leaf litter) surrounded on all sides by the tea gardens of Tamil Nadu. This species was found nesting beneath the marker stone on the border which separates Kerala and Tamil Nadu. It is presumed that the nest was in its initial stages of establishment as there were hardly any galleries and underground chambers. A single queen, 3 ergatoid queens and 7 workers were collected. This species seems uncommon in the Western Ghats range, since it was not encountered again from any other locality.

#### Cerapachys wighti sp. n.

http://zoobank.org/EB5CD657-F22C-4E1B-8352-995242B9531D

http://species-id.net/wiki/Cerapachys_wighti

**Figures 38–40.**
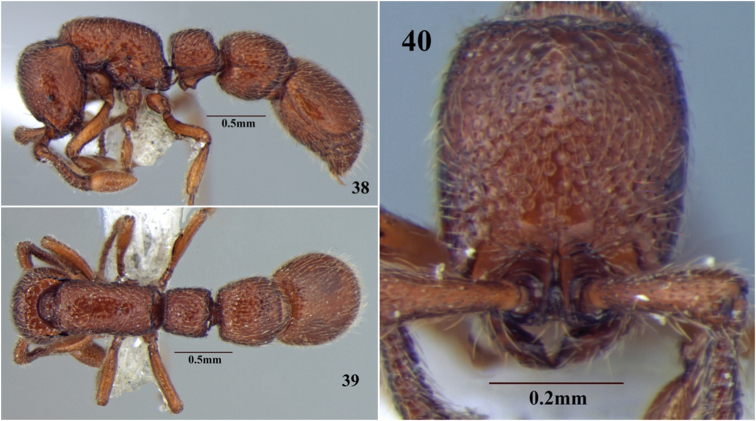
*Cerapachys wighti* sp. n. **38** body in profile **39** body in dorsal view **40** head in full-face view.

Figures 5B , 6B , 9A , 38 , 39 , 40 , Table 1

##### Type material.

Holotype and paratype worker: India. Kerala, Silent valley national park, 11°5'N, 76°26'E, 897m a.s.l., 25.ix.2011, Winkler (coll. Shahid A. Akbar). Holotype in PUAC and paratype in BMNH.

##### Worker description.

Measurements (holotype in brackets): HL (0.69)-0.71; HW (0.58)-0.59; EL (0.05); SL (0.38)-0.40; WL (0.66)-0.70; MH (0.43)-0.47; PrW (0.33)-0.35; PL1 (0.28)-0.29; PW1 (0.32)-0.33; IIIAL (0.41)-0.44; IIIAW (0.47)-0.49; IVAL (0.71)-0.72; IVAW (0.66)-0.68 Indices: CI 83-(84); SI (65)-67; PI 113-(114).

Head rectangular, longer than broad; sides rounding posteriorly, vertexal margin transverse, posterior lateral corners gently rounded, weakly acute. Parafrontal ridges raised, prominent. Eyes reduced. Mandibles subtriangular; masticatory margin without a row of small denticles. Lateroclypeal teeth reduced. Antennae 12 segmented; scapes short, clavate.

Mesosoma stout, compact, rectangular in dorsal view; dorsal surface slightly convex, the sides gently rounded without any distinct margin. Declivous face of propodeum with the upper sides margined.

Petiole broader than long, without strong overhanging dorsolateral margins. Anterior and posterior faces transverse. Subpetiolar process stout with hook like ventral margin; no fenestra present. Postpetiole sub trapezoidal, wider behind, posterolateral angles uniformly rounded. Gaster elongate; base of cinctus of first gastral tergite with cross ribs; sting exerted.

Sculpture. Mandibles punctured. Punctures on dorsum of head large, crowded, their diameter as large, or larger than, the average distance separating them. Mesosoma, petiole and postpetiole similarly sculptured. Gaster with smaller sized punctures compared with head, mesosoma and metasoma. Cinctus of 1^st^ gastral with cross ribs.

Vestiture. Body with reduced pilosity; moderate decumbent or subdecumbent hairs. Mostly prominent on postpetiole and gaster. Apical funicular segments and legs with standing hairs.

Colour. Dark reddish brown with mandibles, antennae and legs lighter

##### Etymology.

The species is named after botanist Robert Wight, who historically explored the area in 1847.

##### Differential diagnosis.

The new species can easily be separated from most of the Indian species on the basis of the large crowded punctureson its cephalic dorsum, with diameter as large, or larger than, the average distance separating them. *Cerapachys wighti* shares most characters with *Cerapachys indicus*, which also has large crowded punctures on cephalic dorsum. However the two species can be easily separated. *Cerapachys wighti* is smaller in size (HW 0.59 mm), has lighter body colouration and reduced eyes (EL 0.05 mm), while *Cerapachys indicus* is larger in size (HW 0.77 mm), with darker body colouration and large eyes (EL 0.24 mm).

##### Ecology.

The species seems to be of rare occurrence as it was encountered only once during the extensive surveys conducted in the area. It was collected from a litter sample taken near the Kuntipuzha river, which drains the entire length of the silent valley national park. With a pesticide free catchment area the region is rich in soil biota and ideal for cryptic ant species.

##### General discussion.

Herewe present a review of genus *Cerapachys* from India. 12 species are recognized of which 6 are described as new. Partly for convenience the 12 Indian species are placed into arbitrary groups. Group I species with 12 segmented antennae viz., *Cerapachys sulcinodis*, *Cerapachys anokha*, *Cerapachys schoedli*, *Cerapachys seema*, *Cerapachys indicus*, *Cerapachys aitkenii*, *Cerapachys wighti*, *Cerapachys longitarsus* and *Cerapachys nayana*. Of the 9 species given above the first four i.e., *Cerapachys sulcinodis*, *Cerapachys anokha*, *Cerapachys schoedli* and *Cerapachys seema*, have the punctures on the dorsum of the head relatively small, separated, with their diameter smaller than the average distance separating them. Among these *Cerapachys anokha*, with the declivous face of the propodeum lacking cariniform margins, and *Cerapachys sulcinodis*, with the dorsal surface of the petiolar node with a smooth, median area are distinct species in the group. *Cerapachys schoedli* and *Cerapachys seema* are easily separated. *Cerapachys seema* has dull body colouration, sculpture much more prominent and coarse, pilosity denser and head almost oval, with the anterior and posterior sections of its sides converging, while *Cerapachys schoedli* is brightly coloured, with sculpture and pilosity reduced and the head rectangular with parallel sides. The next 3 species i.e., *Cerapachys indicus*, *Cerapachys aitkenii* and *Cerapachys wighti*, have the punctures on the dorsum of the head large, their diameter greater than the average distance separating them. Among these *Cerapachys wighti* has the smallest size (HW 0.59 mm) and relatively reduced eyes (EL 0.05 mm) wereas *Cerapachys aitkenii* and *Cerapachys indicus* are easily separated from each other on the basis of body sculpture and colouration. *Cerapachys aitkenii* has characteristic bicolouration and its body sculpture is foveate, wereas *Cerapachys indicus* is mostly piceous with bluish iridescent sheen and reduced sculpture. The remaining 2 species i.e., *Cerapachys longitarsus* and *Cerapachys nayana* are members of ‘*Phyracaces* lineage’ and easily recognized, with strong overhanging dorsolateral margins to the petiole. The two species are separated from each other on the basis of body colouration. *Cerapachys longitarsus* has characteristic bicolouration with head brown, trunk red or brown, petiole and postpetiole light to dark reddish and gaster brown or black, while *Cerapachys nayana* is uniformly black in colour, with mandibles, antennae and legs castaneous. Group II species have antennae with less than 12 segments viz., *Cerapachys biroi*, *Cerapachys alii* and *Cerapachys besucheti*. Among these *Cerapachys besucheti* has 11 segmented antennae while *Cerapachys biroi* and *Cerapachys alii* have 9 segmented antennae. *Cerapachys biroi* is characterized by its opaque body with closely spaced piligerous punctures, while *Cerapachys alii* has prominent foveate body sculpture.

**Figure 41.**
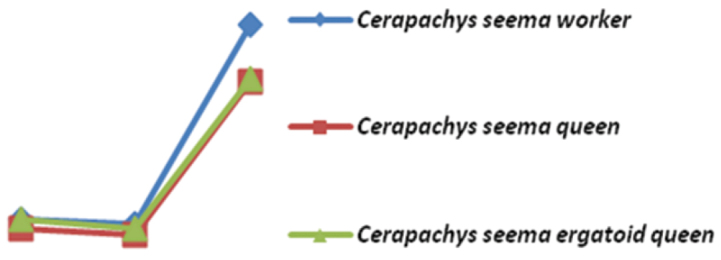
Graph plotted on evaluating morphometric data shows less affinity of ergatoid queens with workers compared with gyne/queen.

Workers grade into a number of “atypical” reproductives. These morphologically “atypical” ant reproductives have been assigned a number of descriptive terms. However Peeters (2012) advocate use of “ergatoid queens” for all wingless reproductives that differ morphologically from workers. These ergatoid queens are formed as a response to selective pressures against long range dispersal and solitary colony foundation (Peeters and Molet 2010). Ergatoid queens have been reported previously in *Cerapachys* (Brown, 1975). Here we present ergatoid queens of three more species - *Cerapachys nayana*, *Cerapachys schoedli* and *Cerapachys seema*. In evaluating morphometric data of the three castes of *Cerapachys seema* i.e. worker, ergatoid queens and queen castes (Fig. 41) it is observed that ergatoid queens are closer to gynes than the workers. Further inference and analysis on the subject is beyond the scope of this paper and would require much more information. However the aim of this review is to add further material for examination for understanding this fascinating aspect of ant biology and to promote more studies in this direction.

##### Notes.

*Cerapachys keralensis* Karmaly, 2012 described on the basis of two minor? workers collected from the Palakkad district of Kerala. The new species is highly dubious. The description is minimal, superficial and contains no comparative notes. The photographs are derisory as illustrations supporting the inadequate descriptions. *Cerapachys keralensis* Karmaly, 2012 is here considered to be a species inquirenda.

Two unpublished new species (*Cerapachys browni* and *Cerapachys costatus*; Bharti and Wachkoo (in press)) are excluded from this paper. The two species can be easily separates from other reported Indian species. *Cerapachys browni* shares mostaffinitieswith *Cerapachys aitkenii* but with black colour (unicolorous), rugo-reticulate sculpture and strongly constricted cintus of gaster. *Cerapachys costatus* with remarkable costate sculpture, which is not reported in any other Indian species.

## Supplementary Material

XML Treatment for Cerapachys alii

XML Treatment for Cerapachys anokha

XML Treatment for Cerapachys nayana

XML Treatment for Cerapachys schoedli

XML Treatment for Cerapachys seema

XML Treatment for Cerapachys wighti

## Appendix 1

Supplementary File 1 of image

## Appendix 2

Supplementary File 2 of image

## Appendix 3

Supplementary File 3 of image

## Appendix 4

Supplementary File of morphometrye

## Appendix 5

Table-1

## Appendix 6

Figure 43
