# Supplementary figures and images for: Taxonomic studies on the ant genus Cerapachys Smith (Hymenoptera, Formicidae) from India
Source: Zookeys. 2013 Sep 27;(336):79–103. doi: 10.3897/zookeys.336.5719 (PMC3800781; doi:10.3897/zookeys.336.5719)

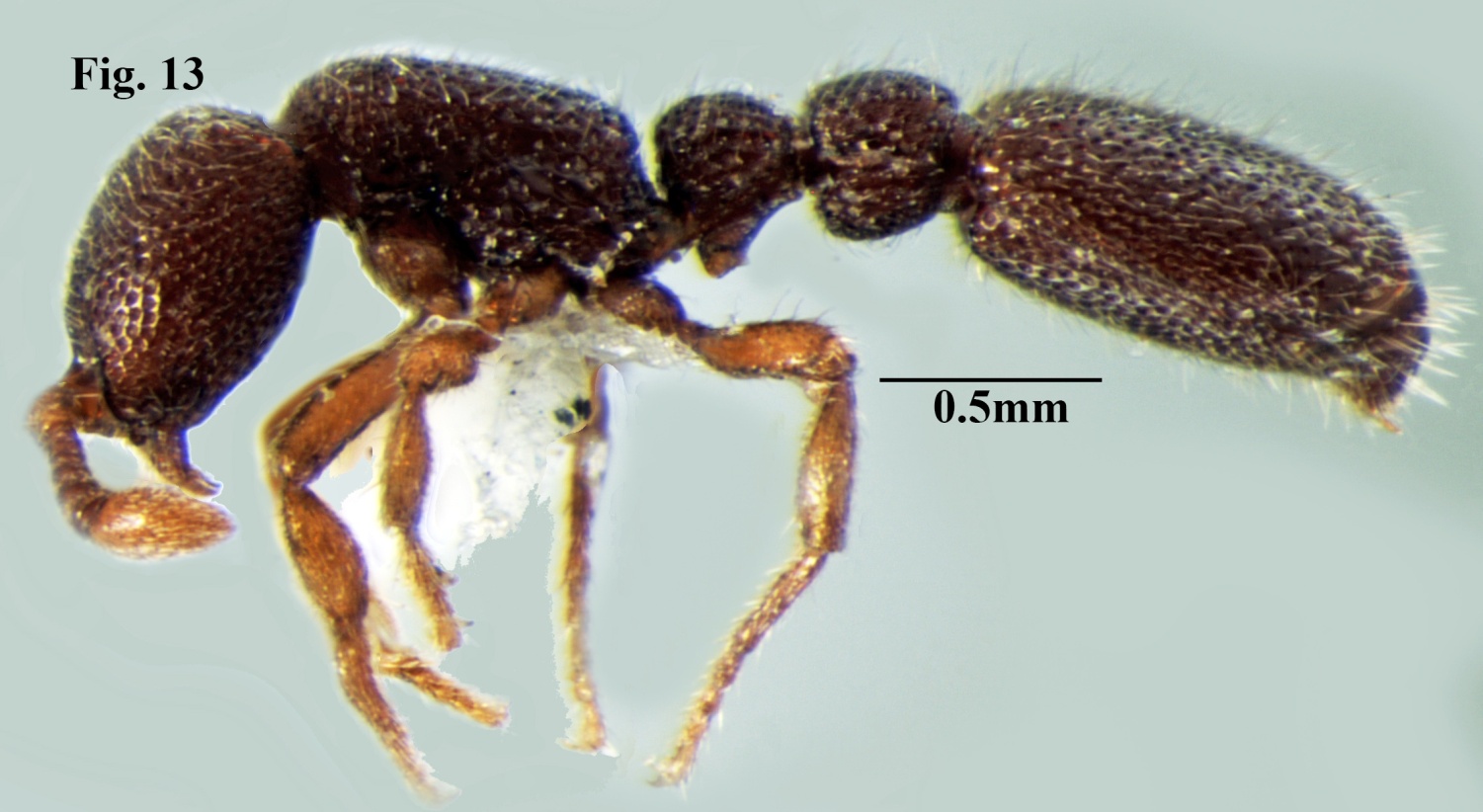


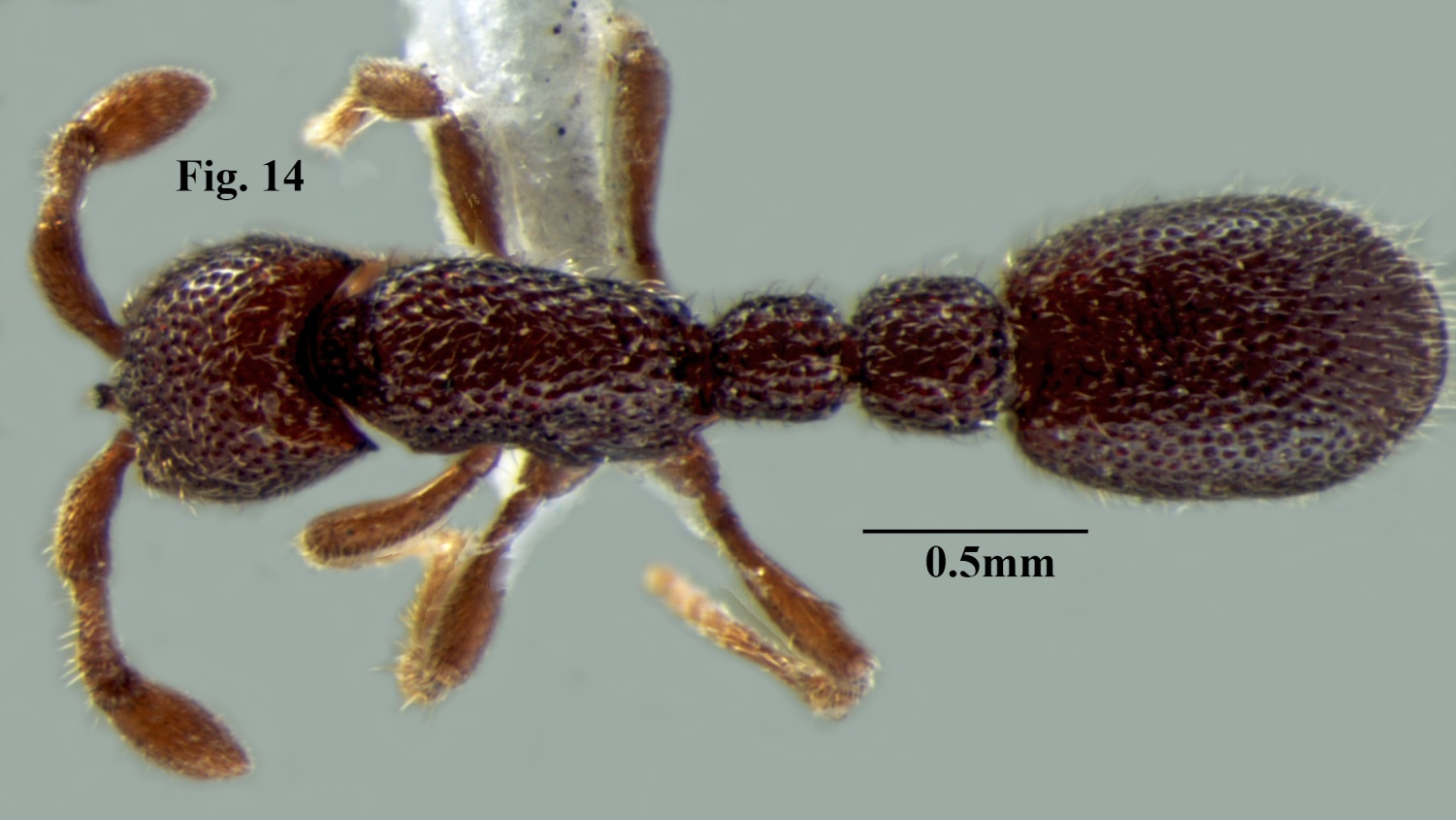


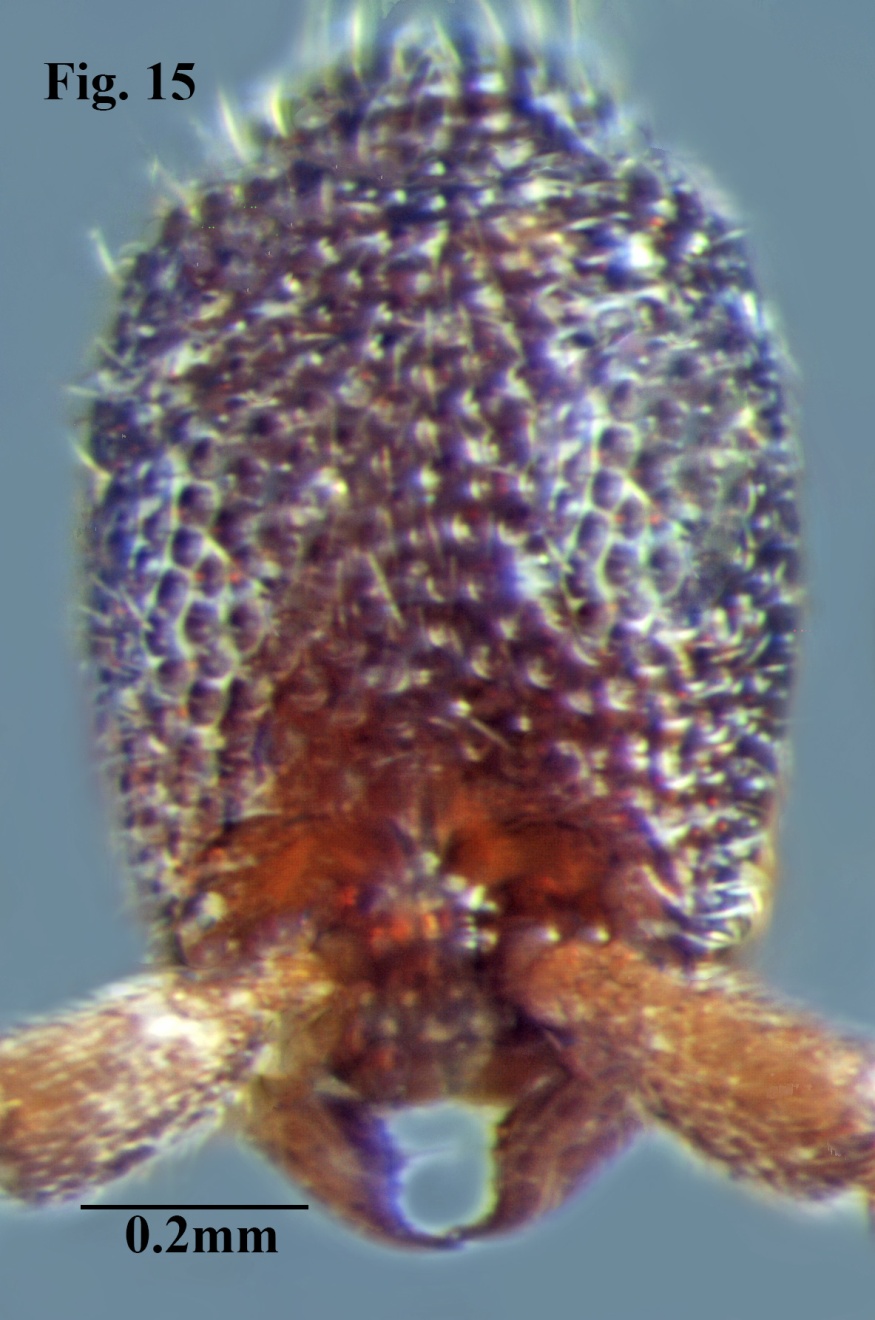


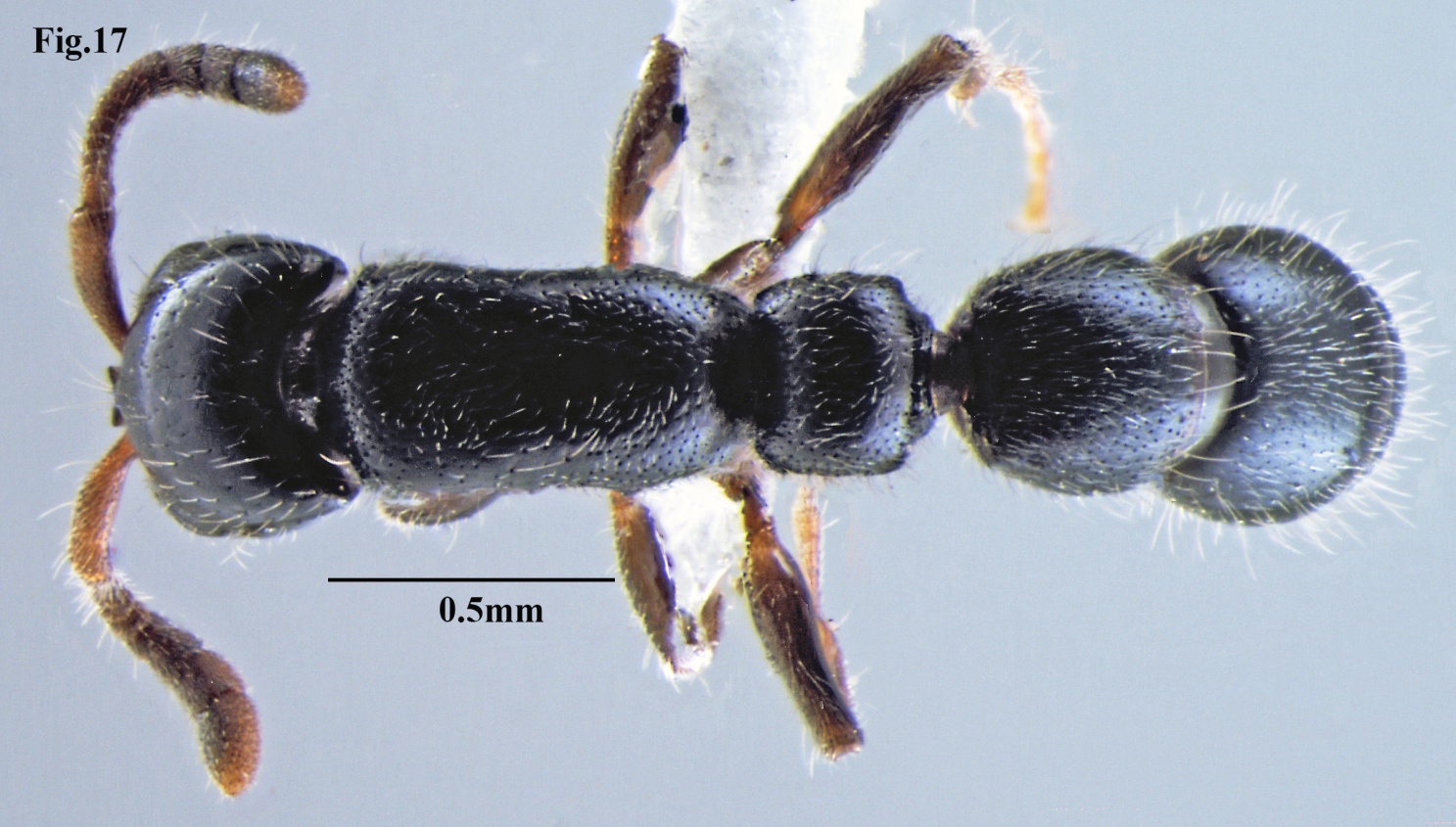

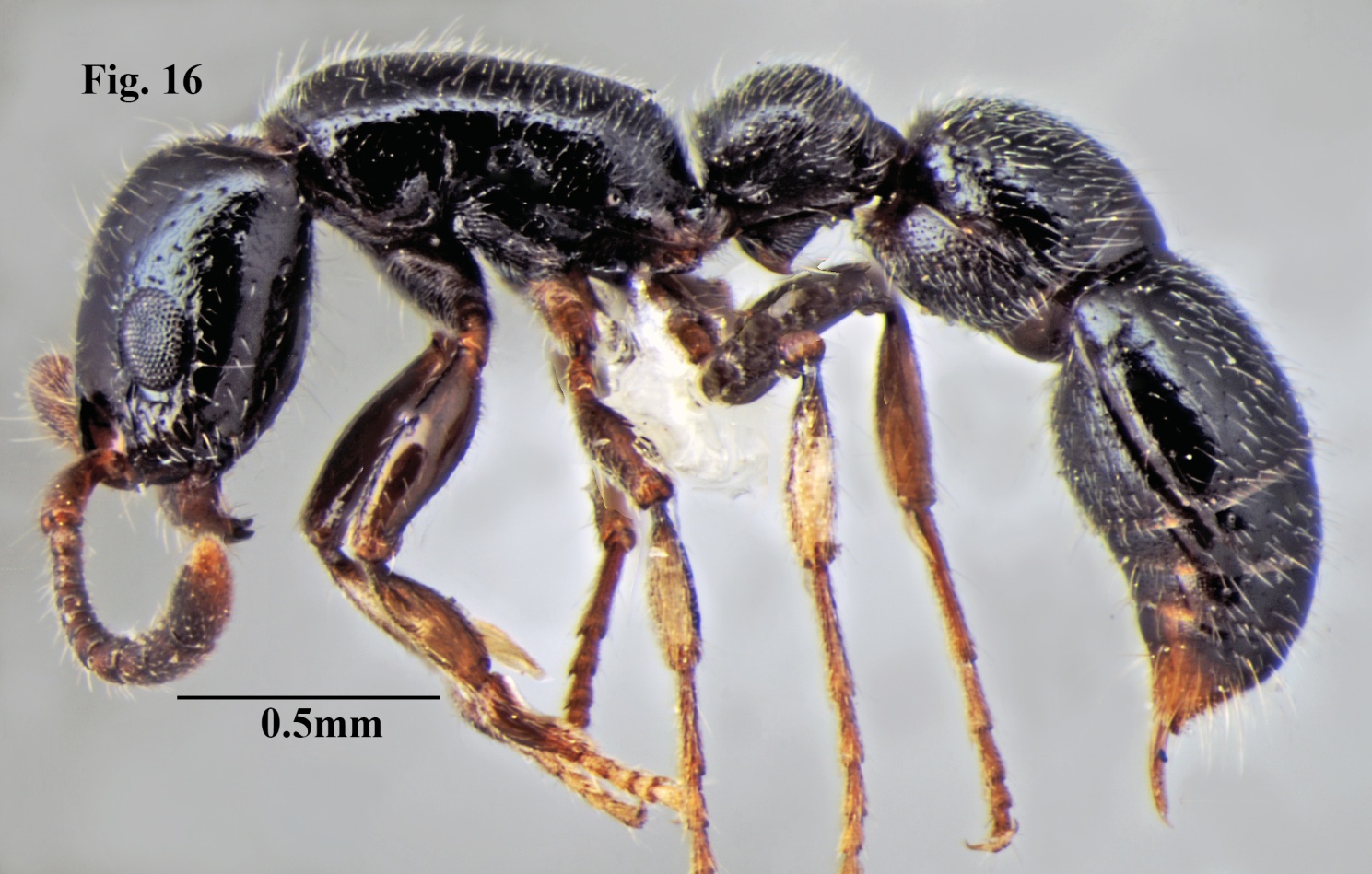


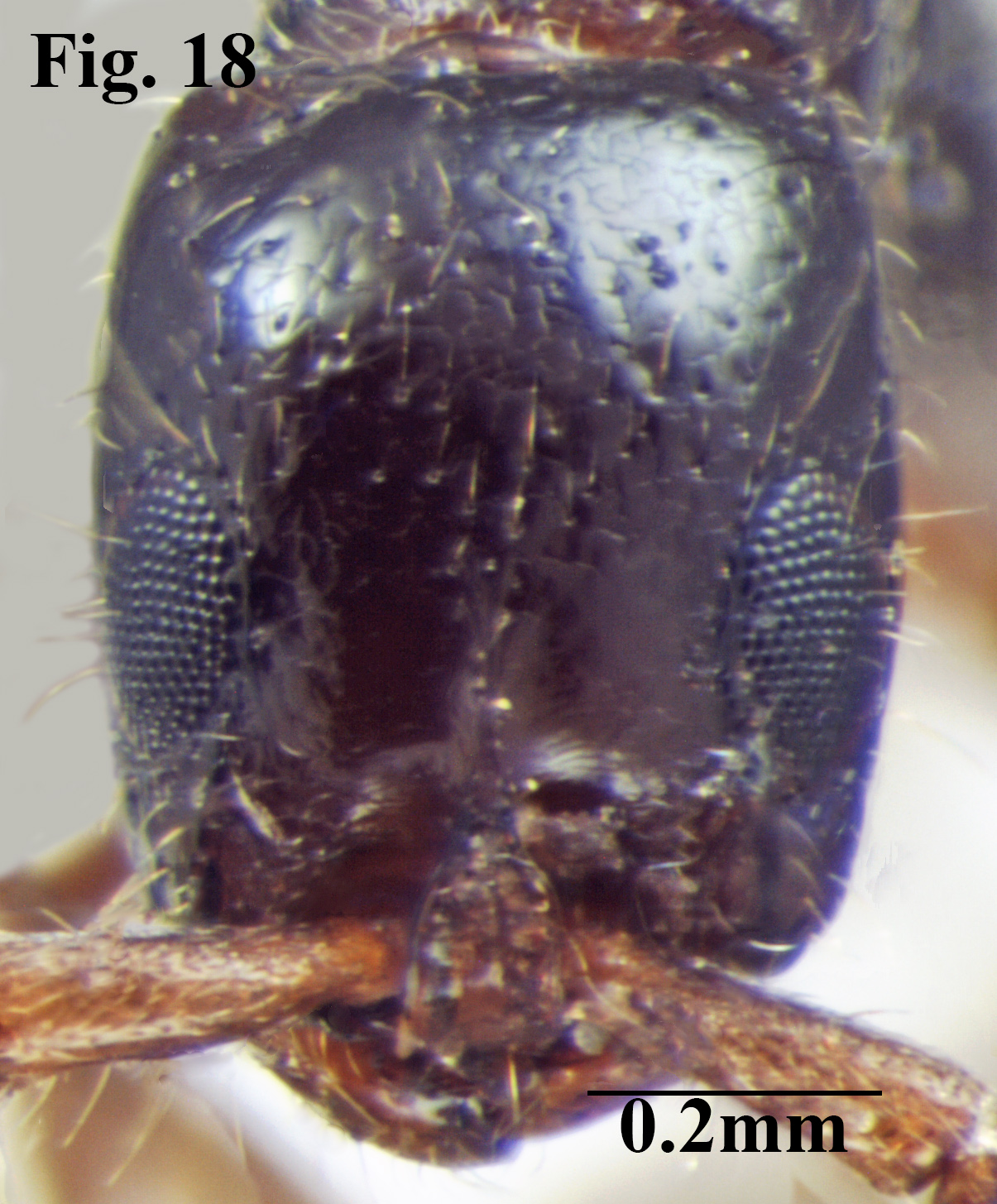


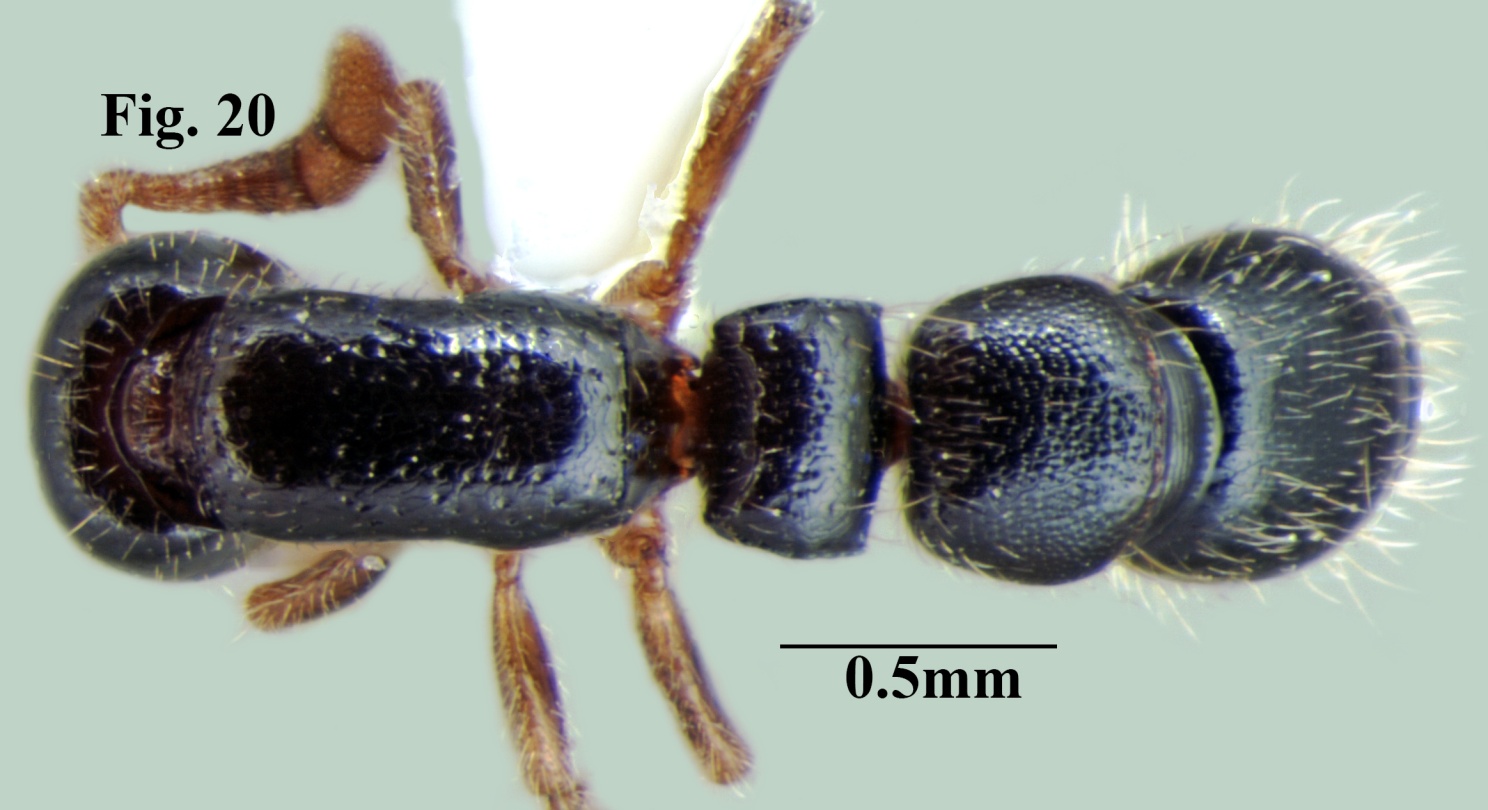

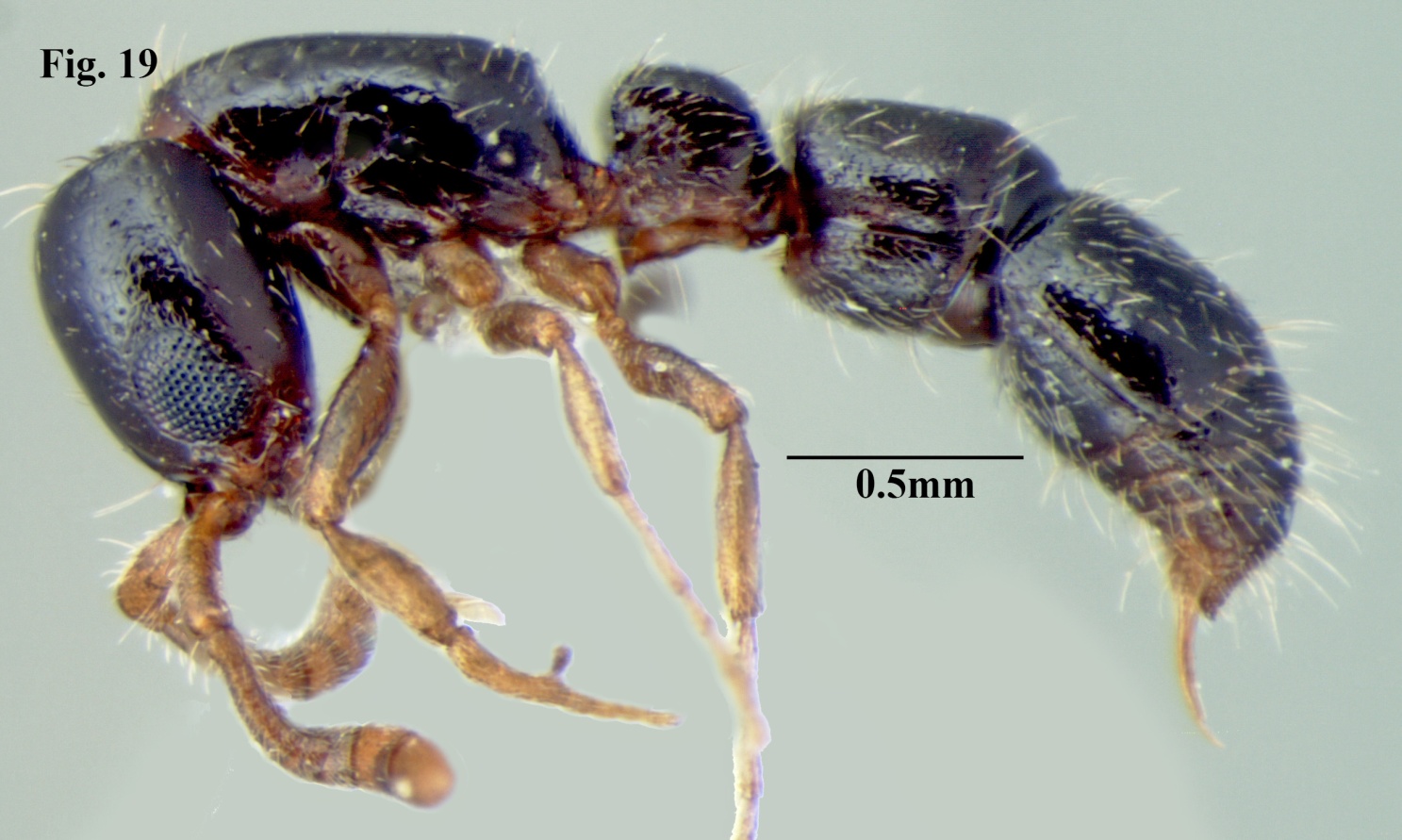


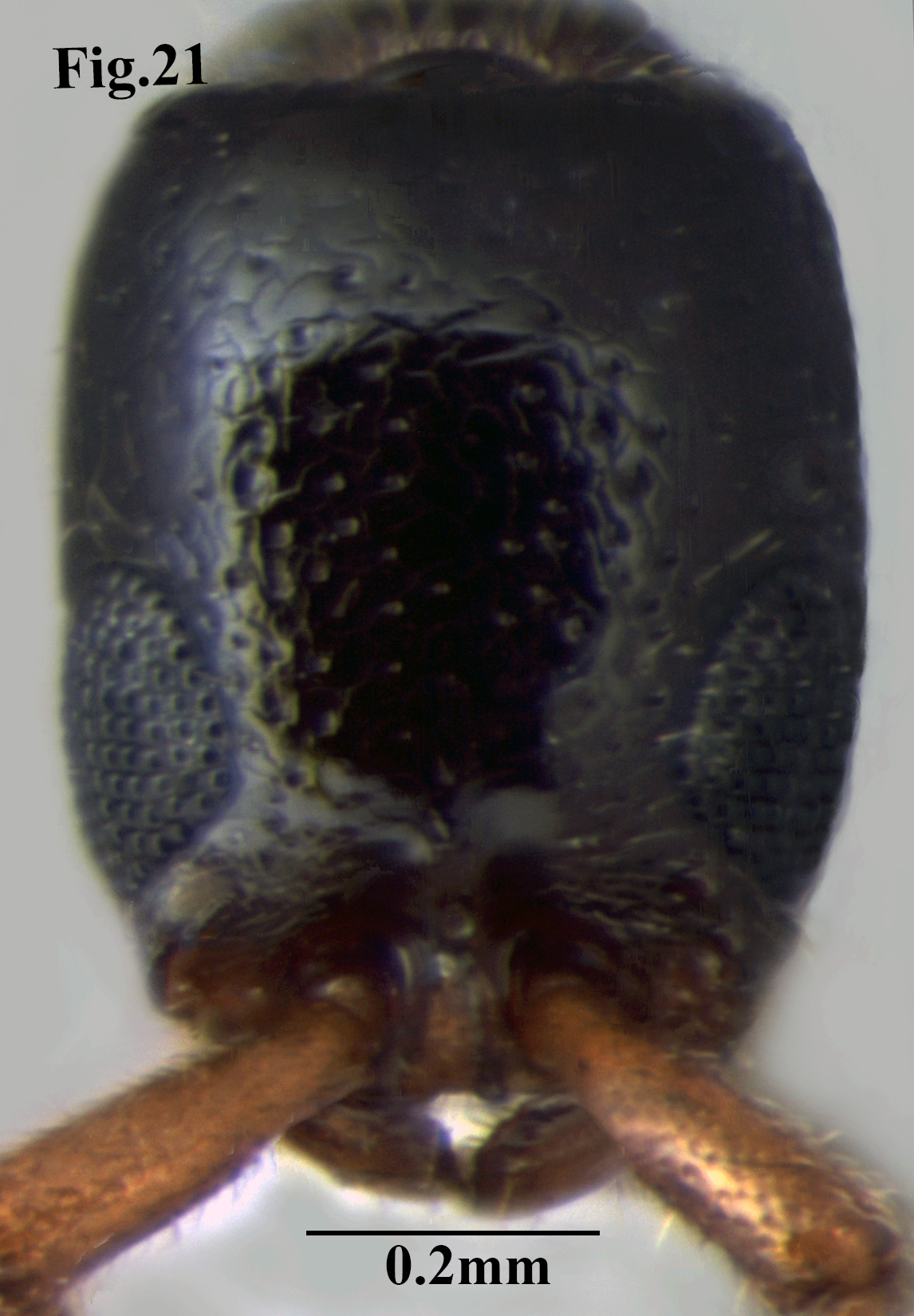


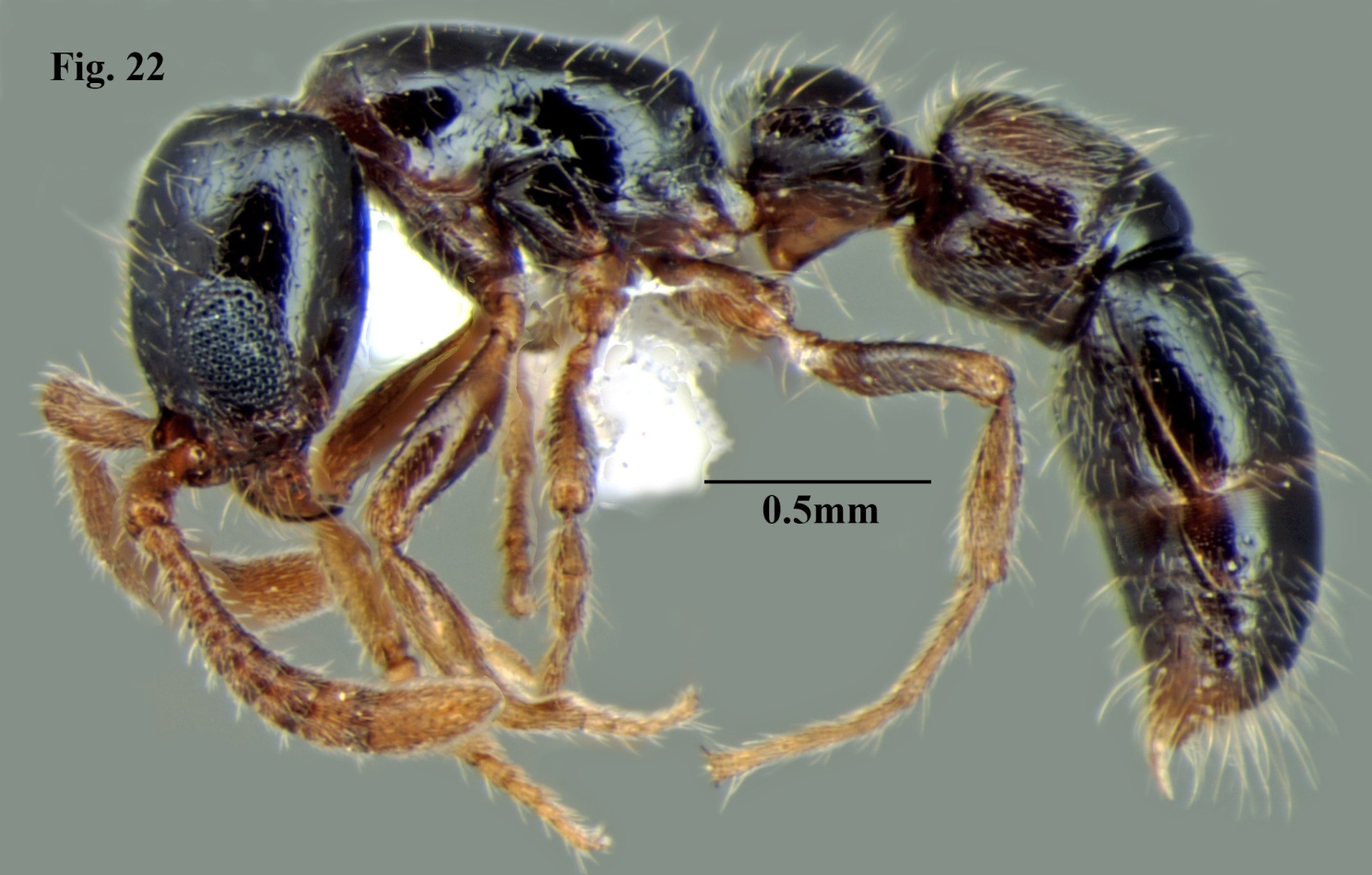


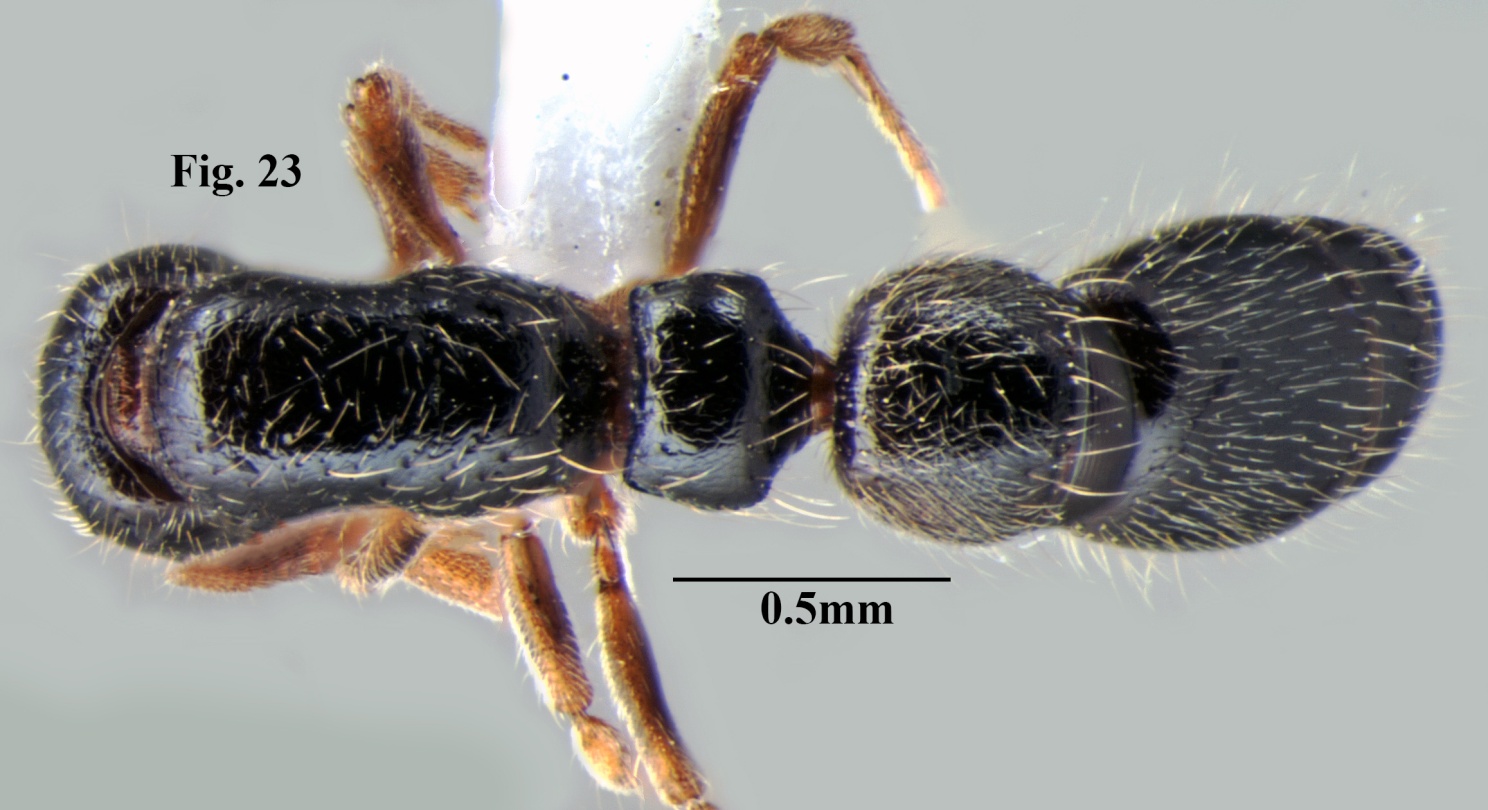


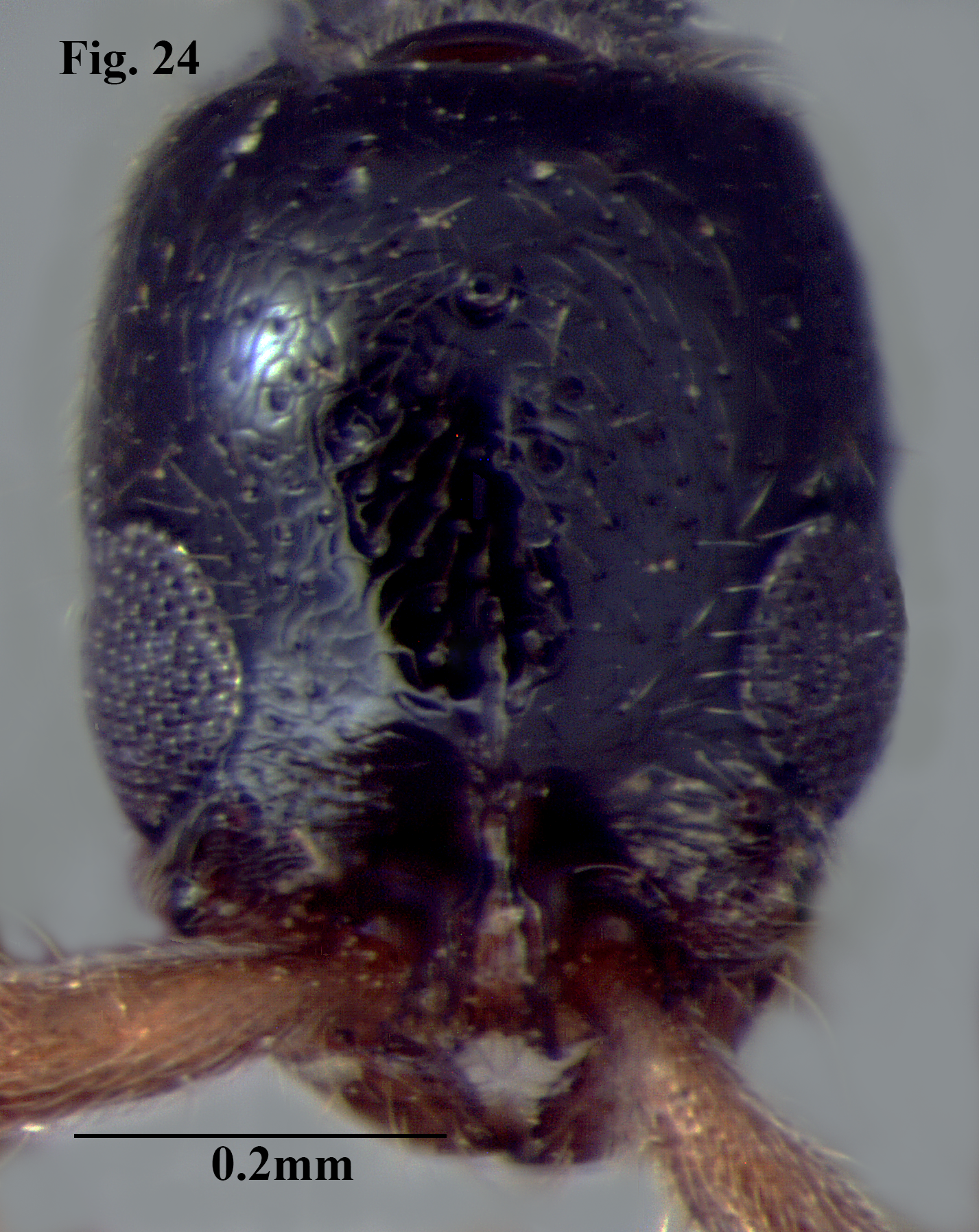

Supplement: Supplementary file 7 — Supplementary File 1 of image [file ZooKeys-336-079-s001.docx]

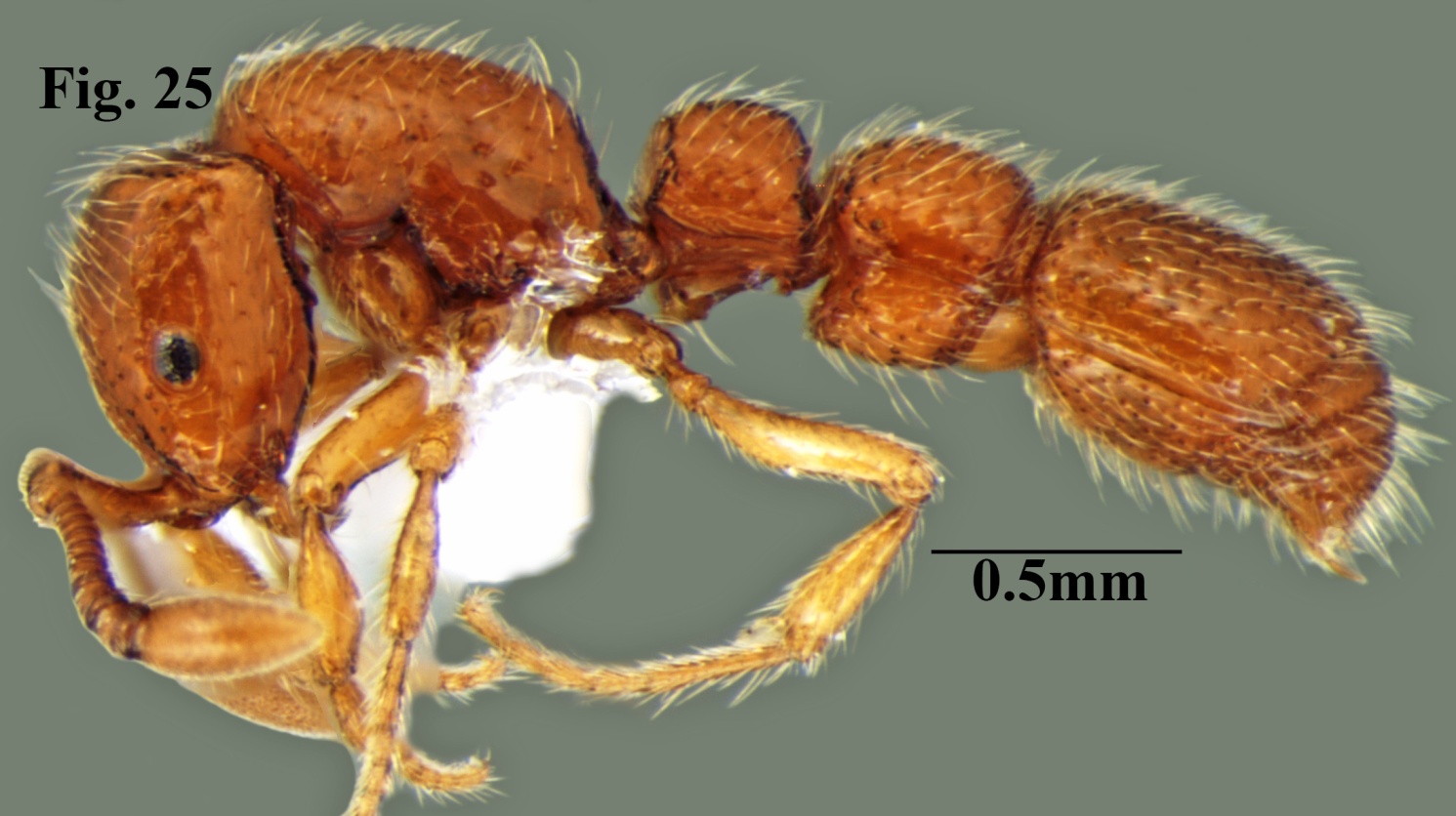

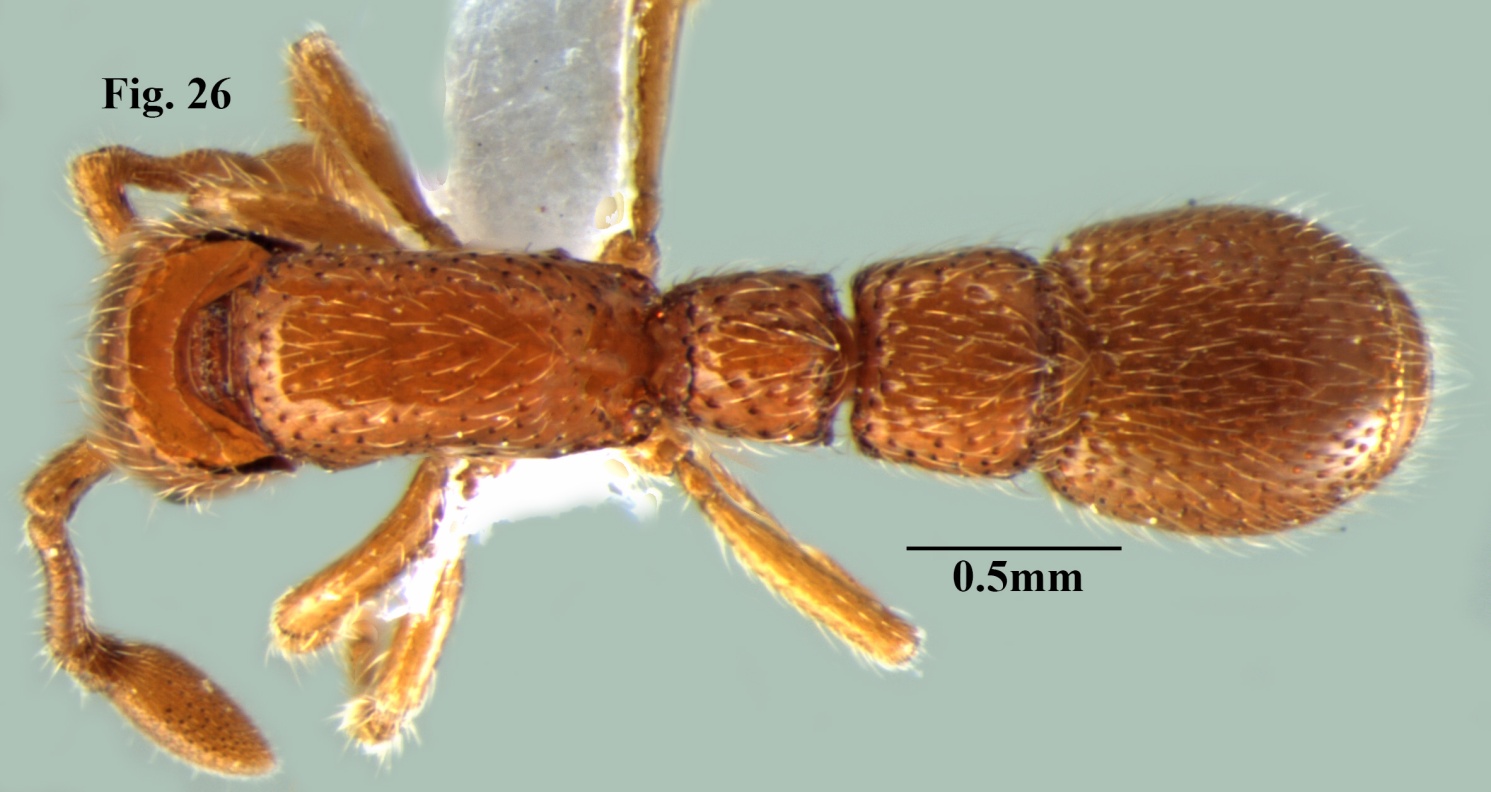


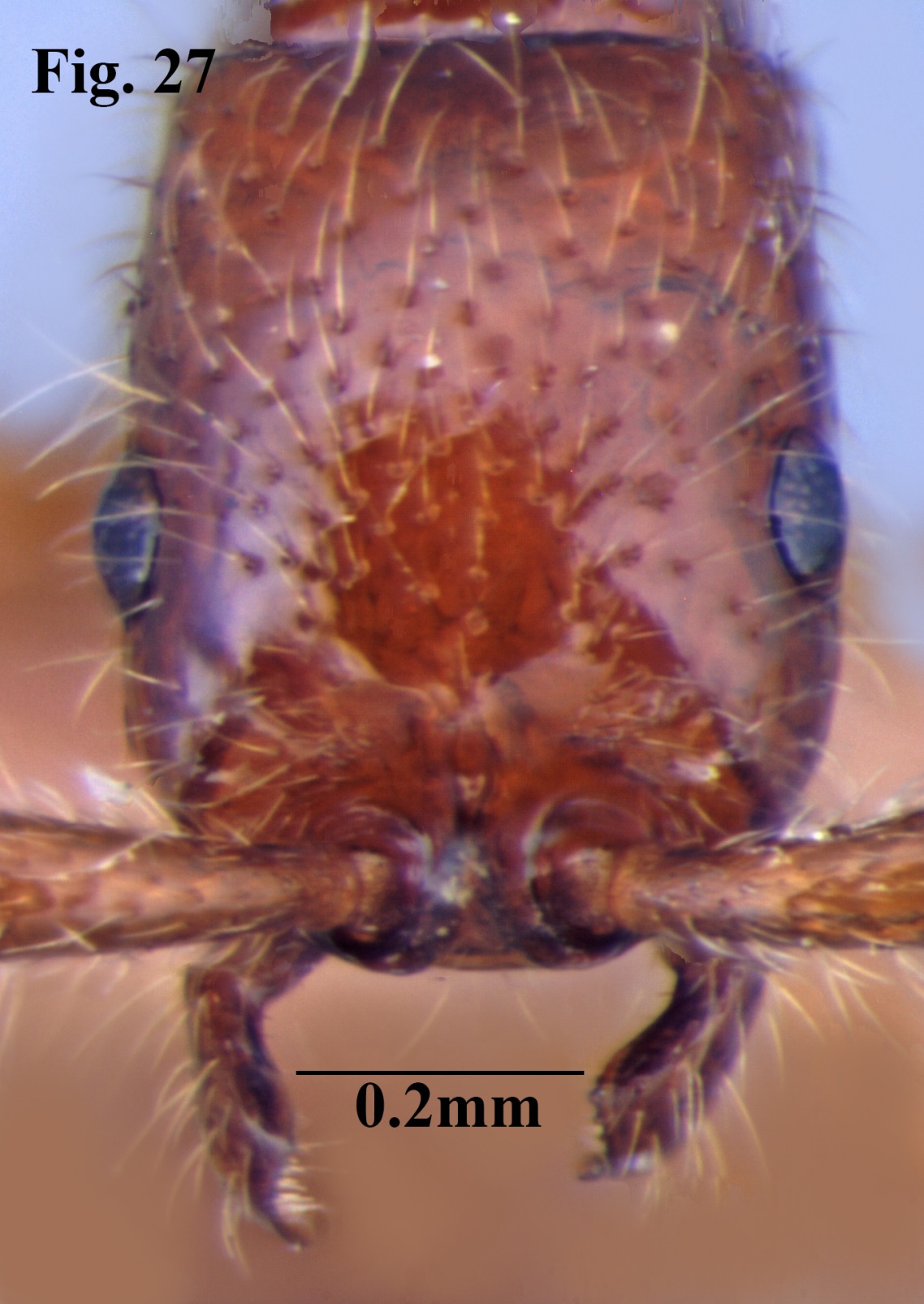


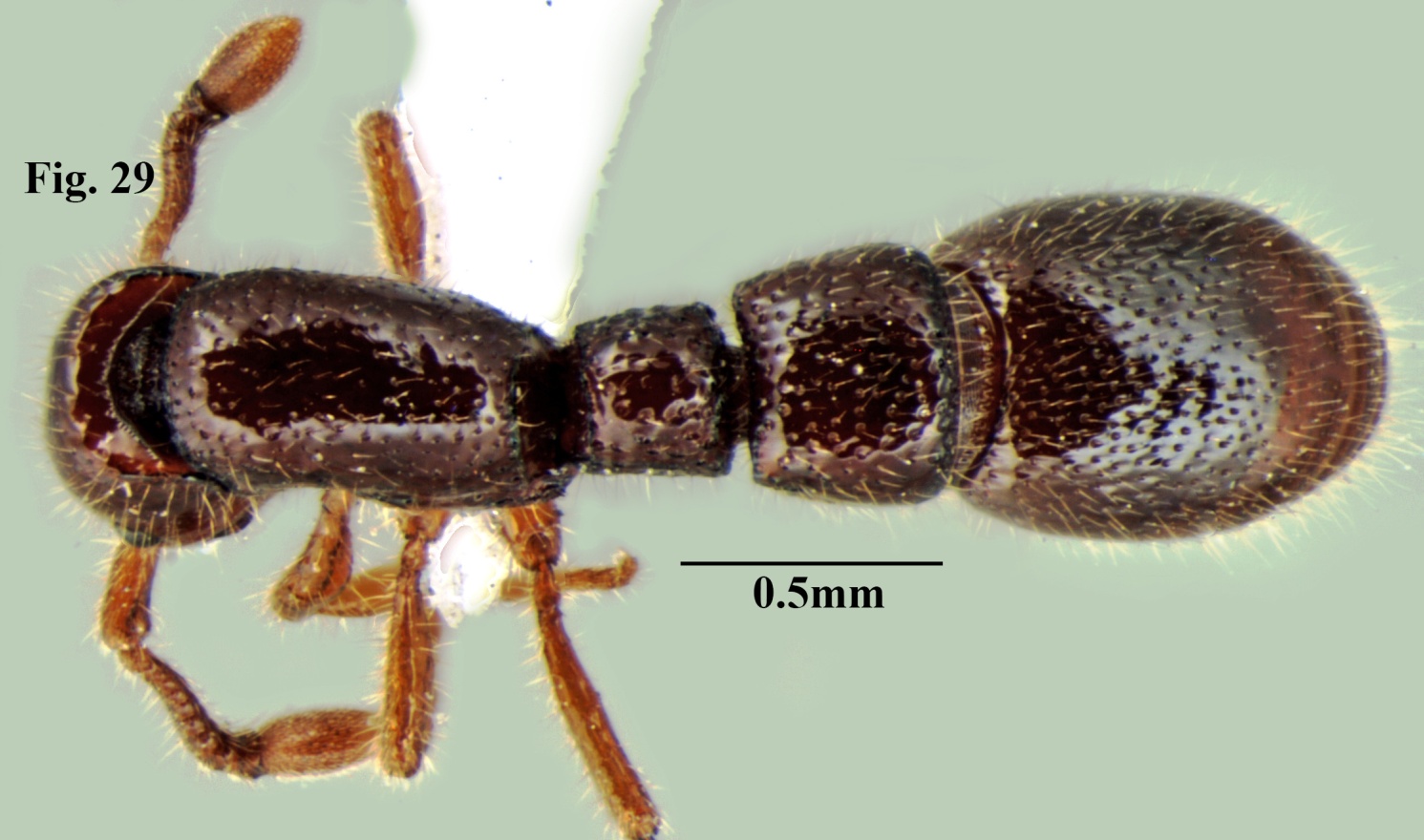

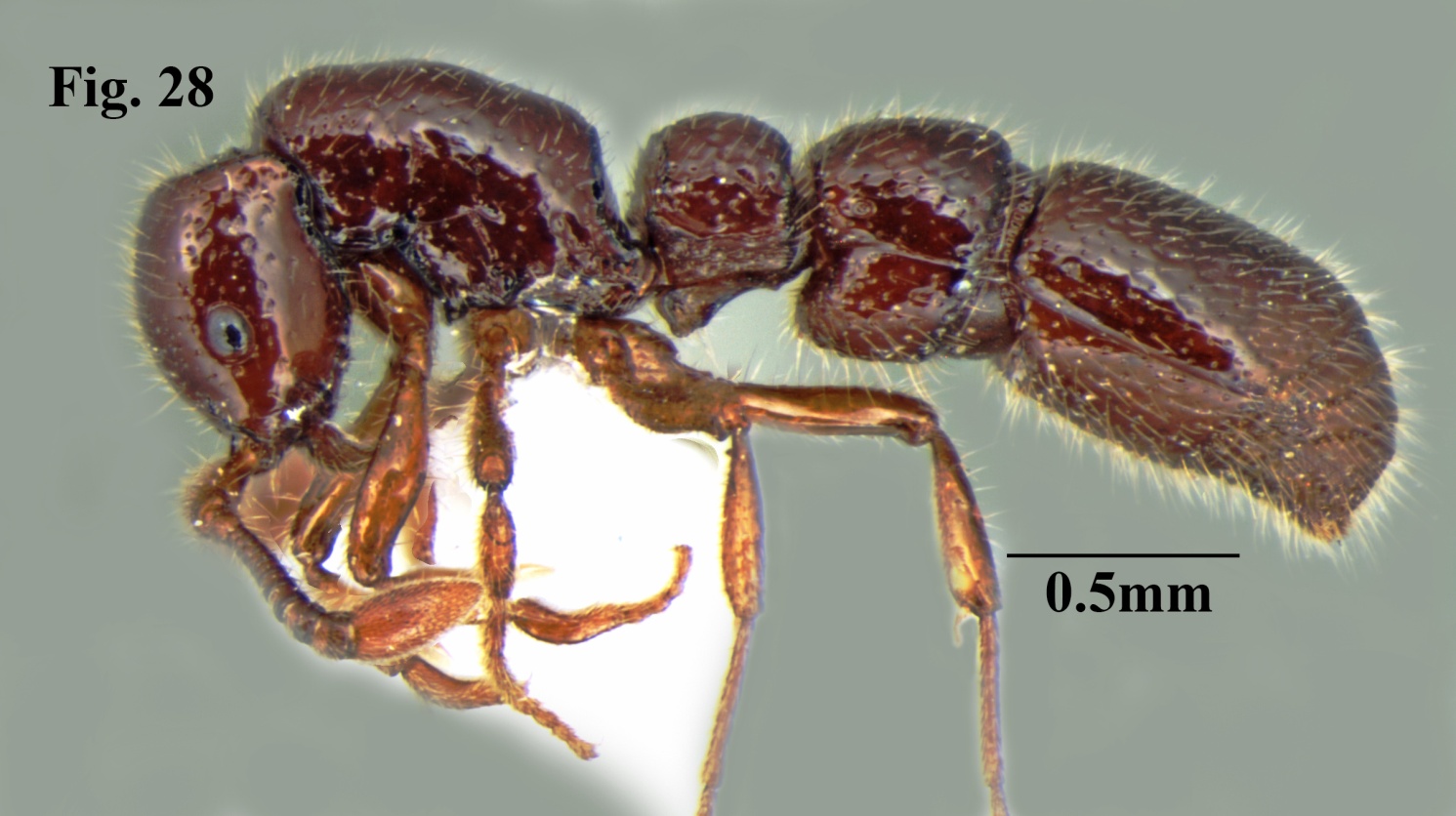


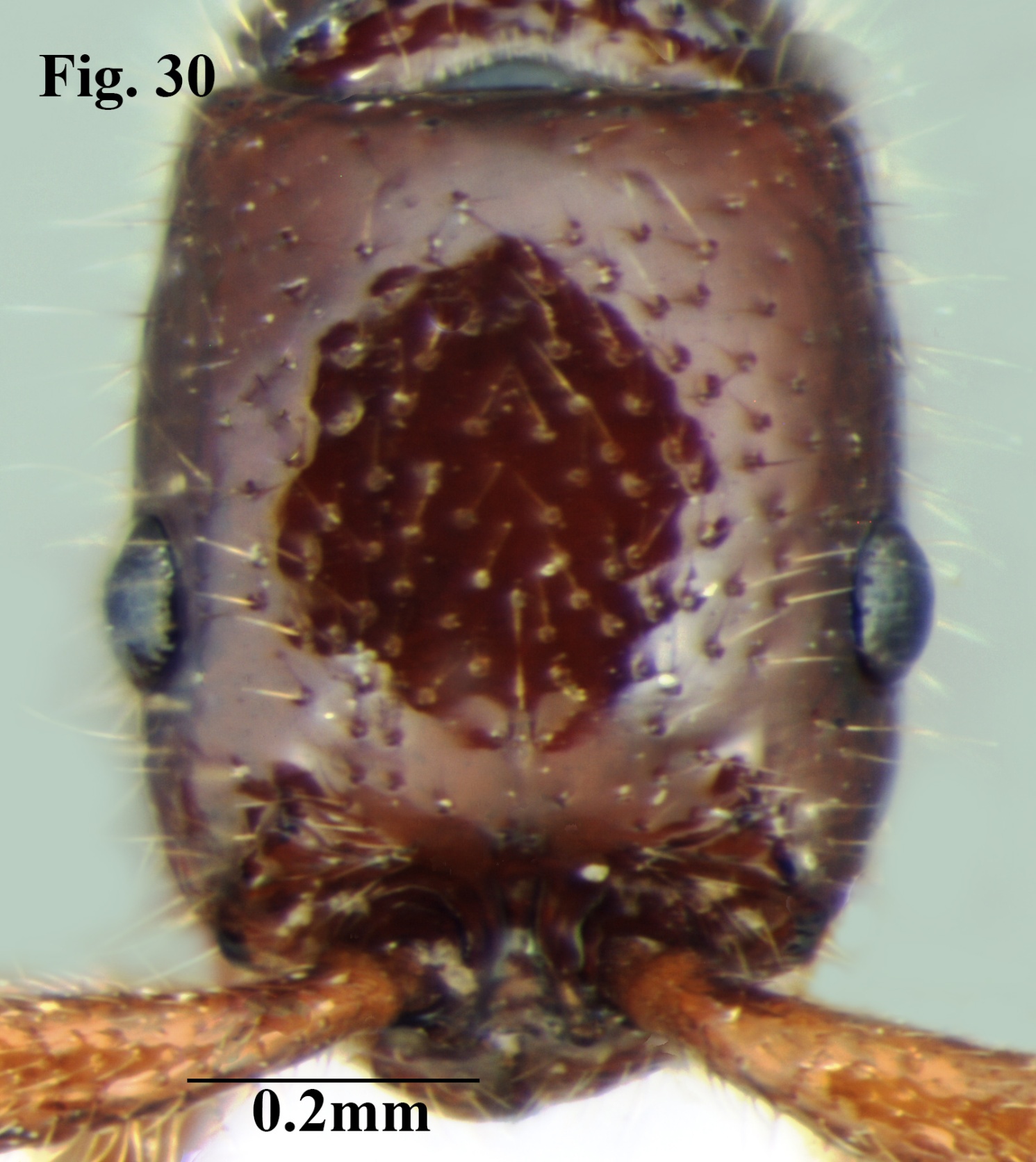


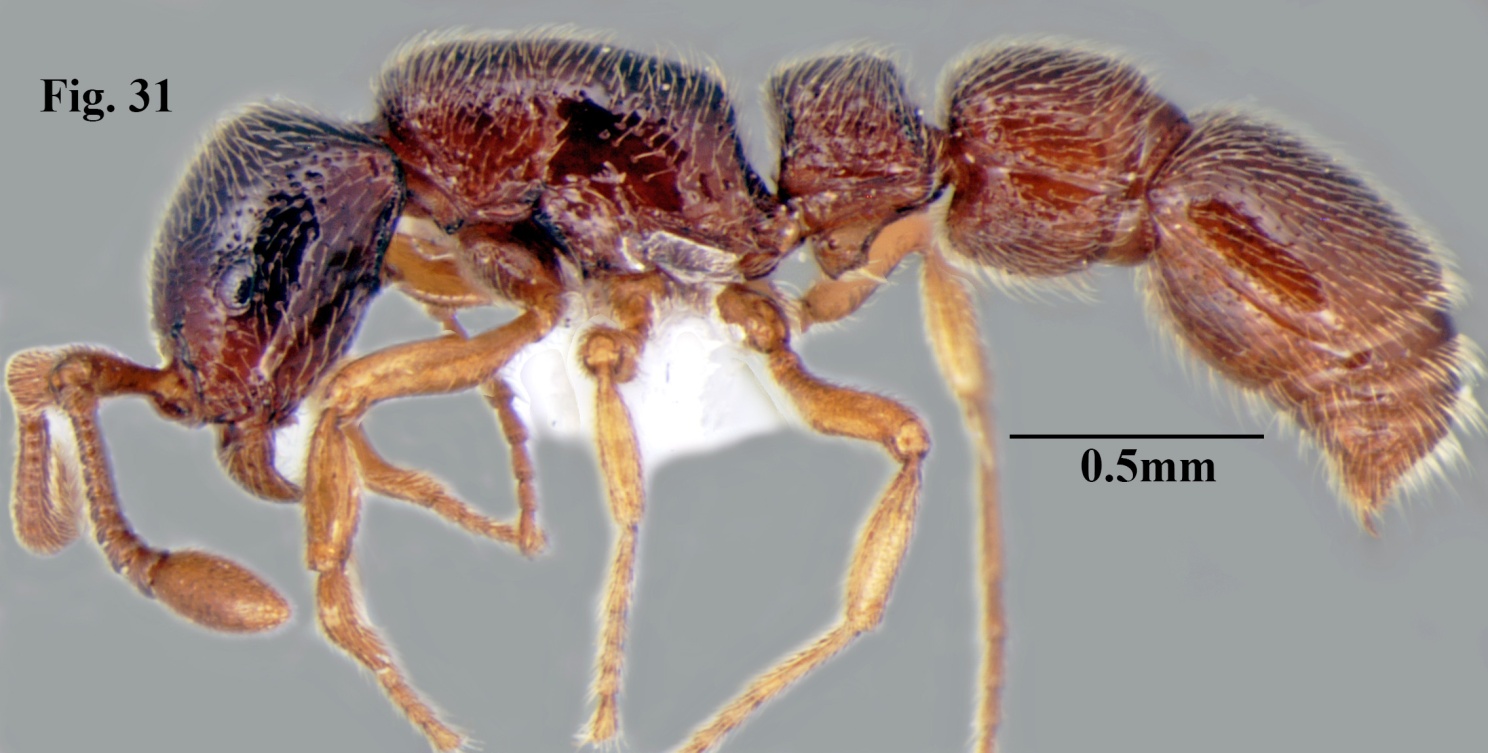

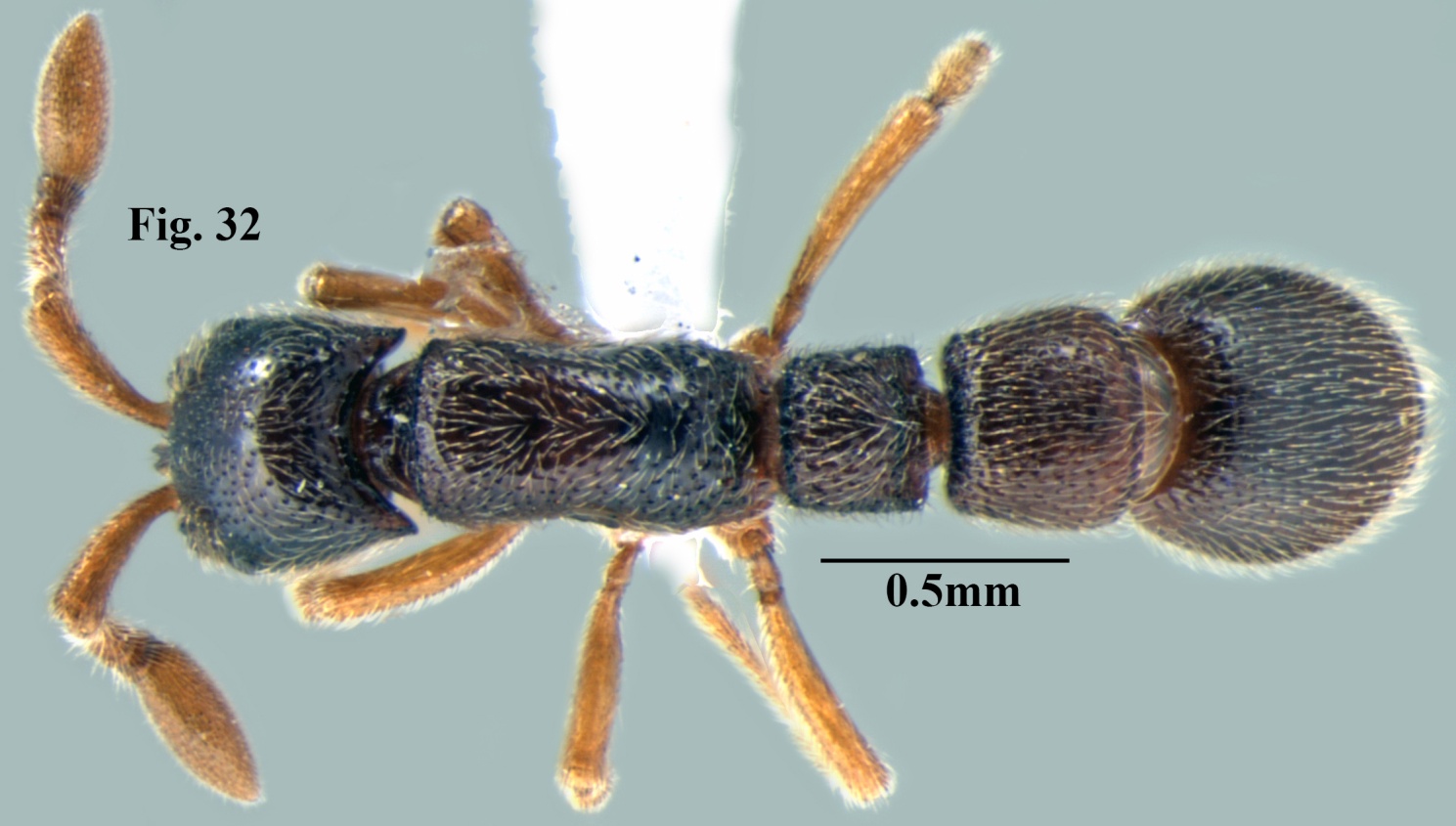


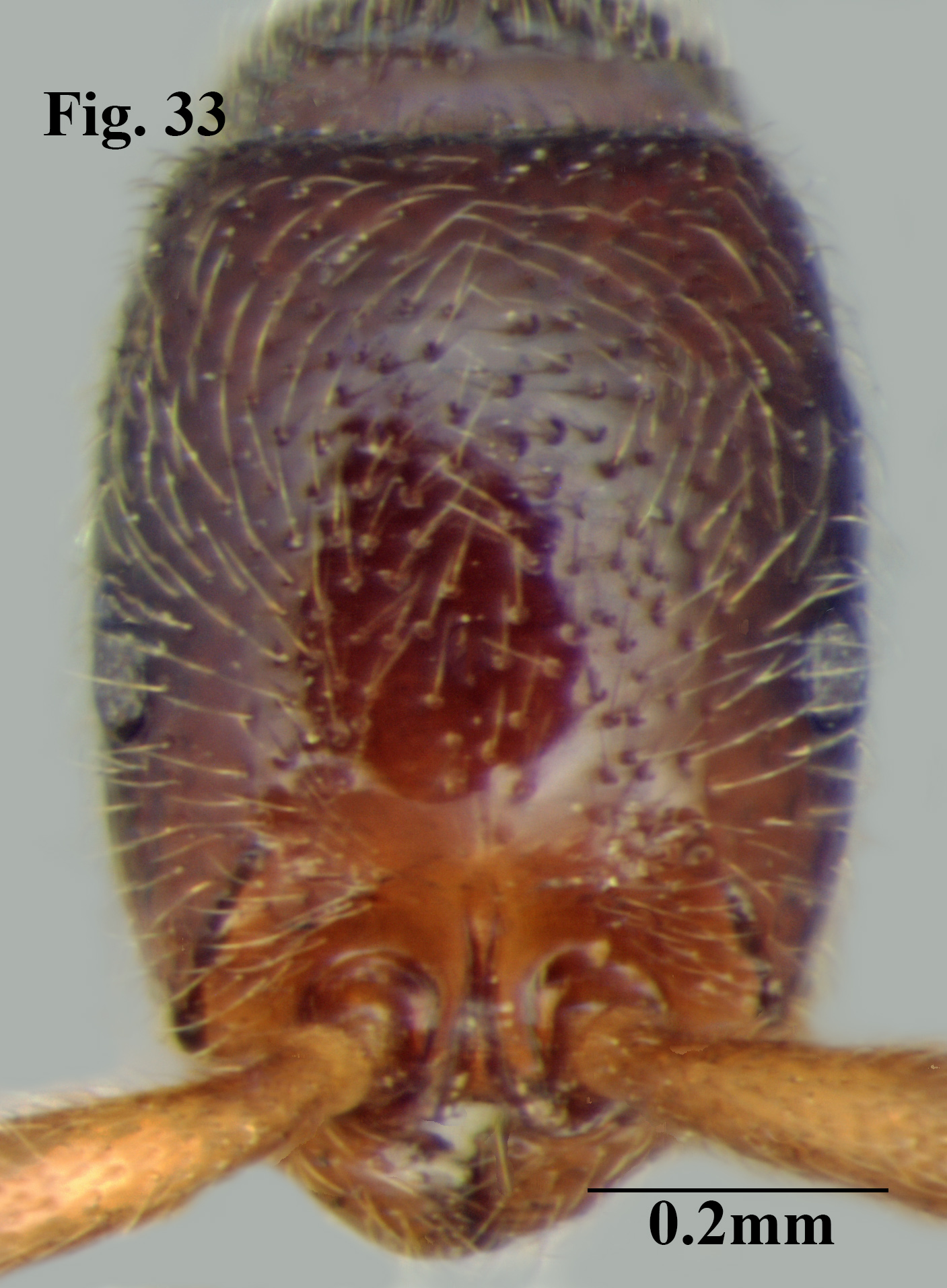

Supplement: Supplementary file 8 — Supplementary File 2 of image [file ZooKeys-336-079-s002.docx]

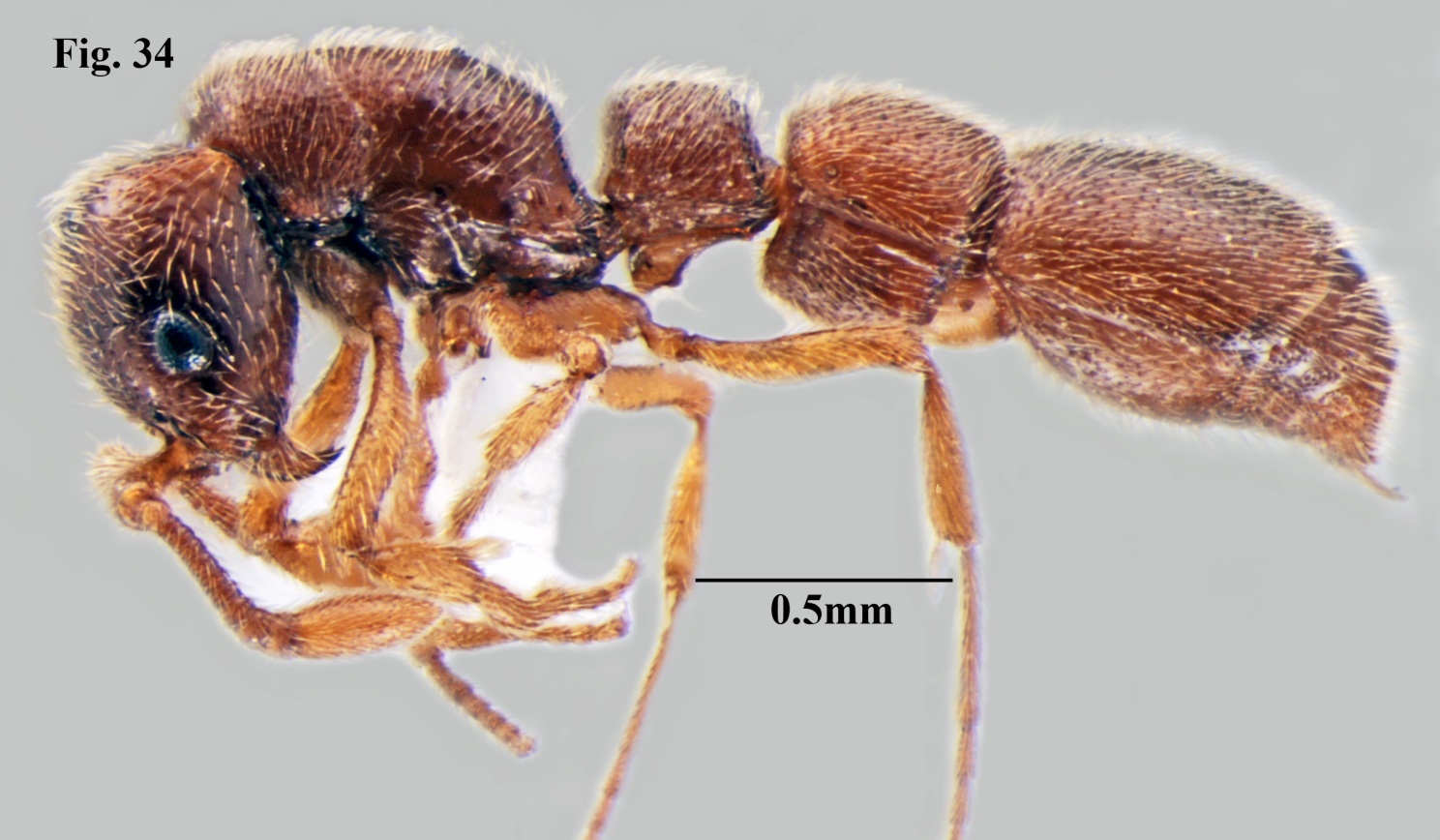


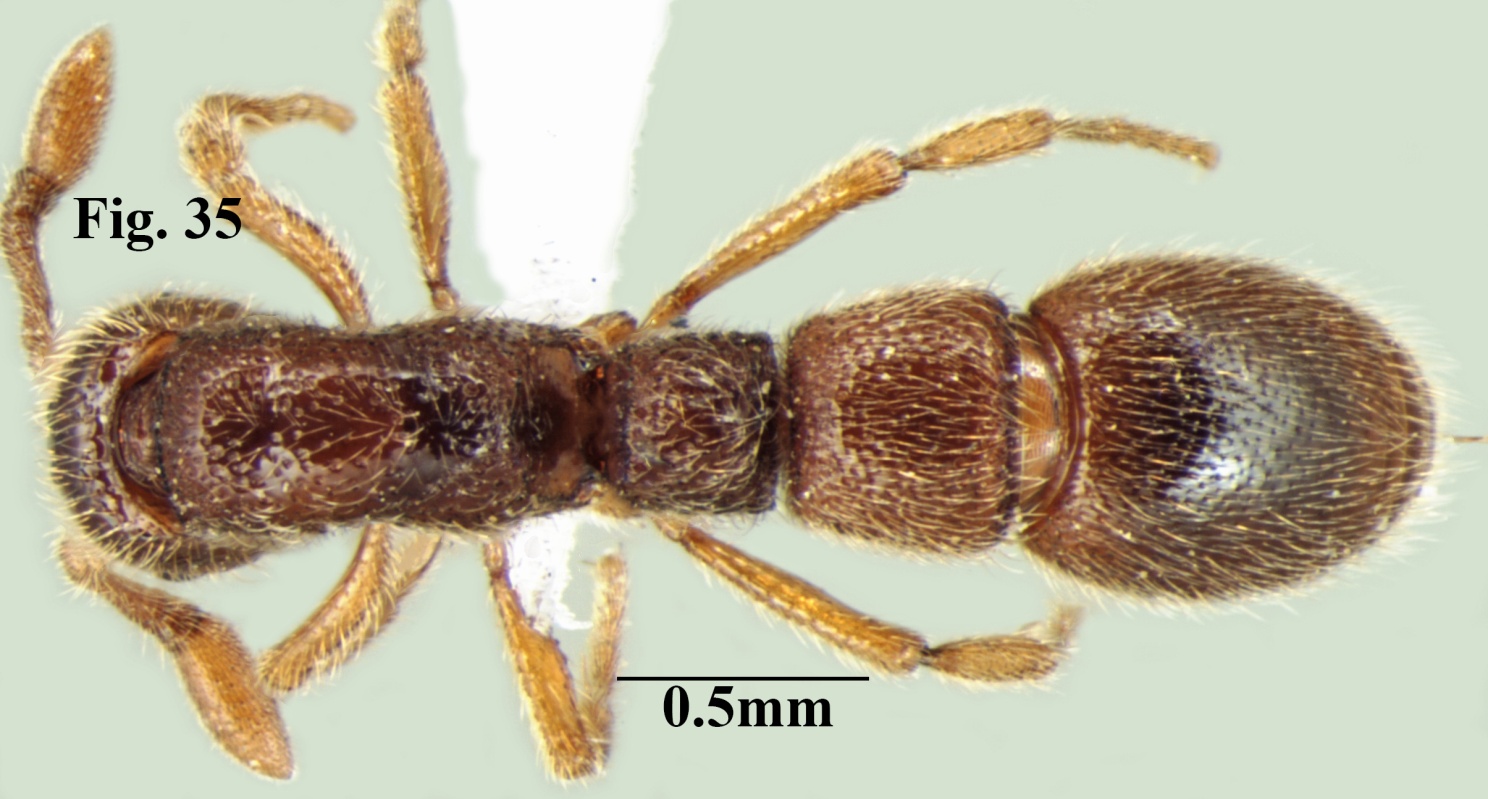


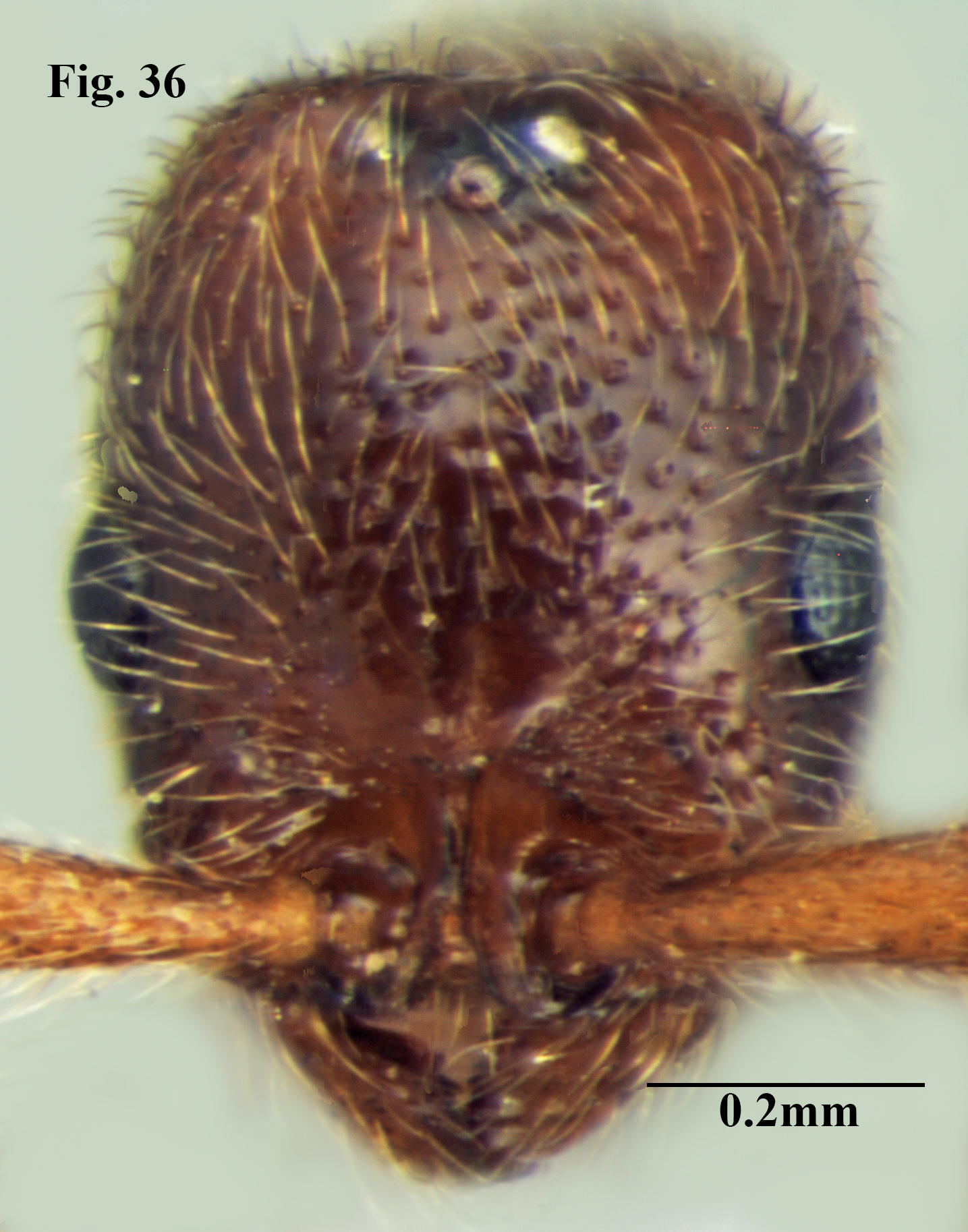


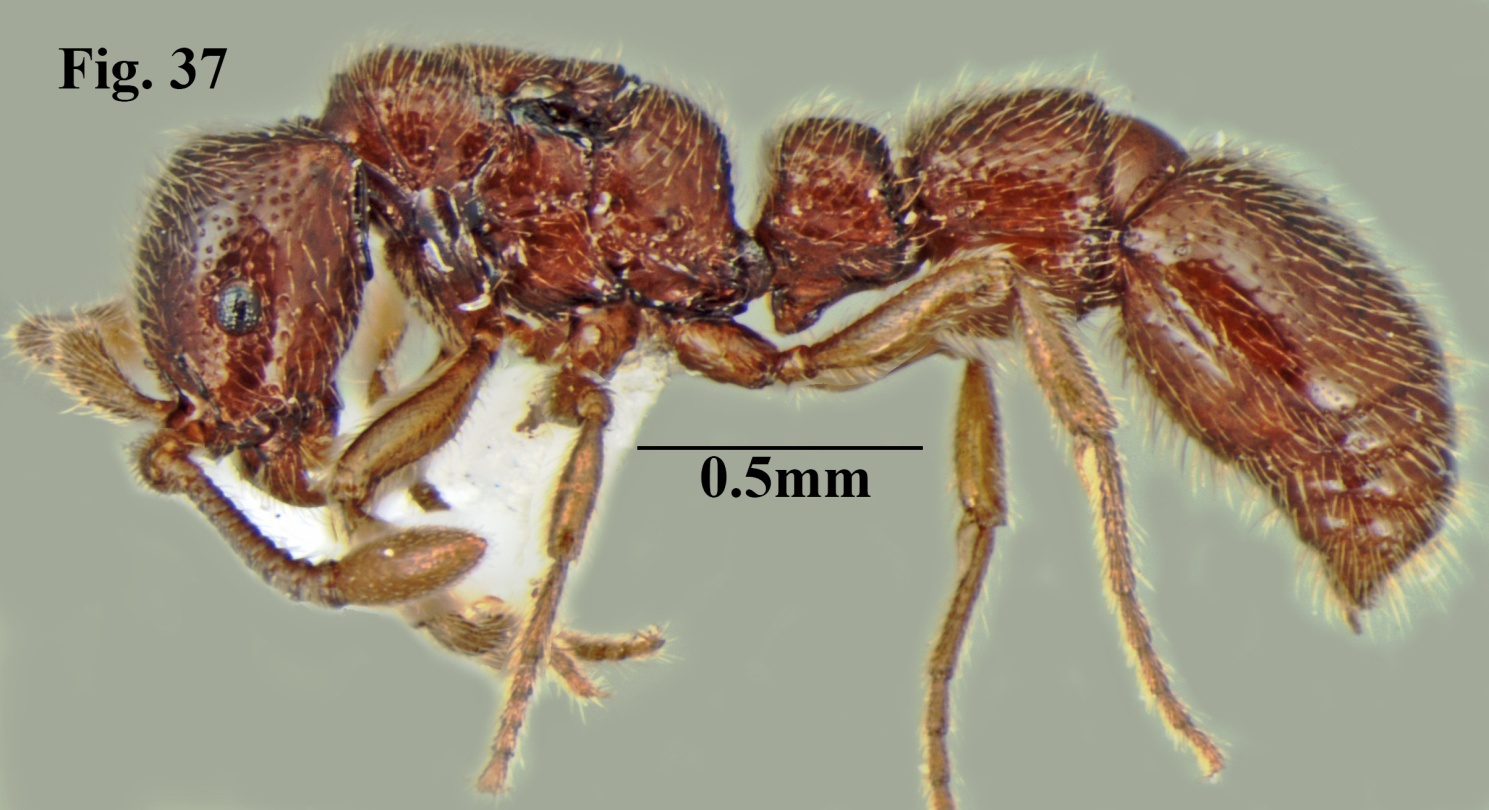


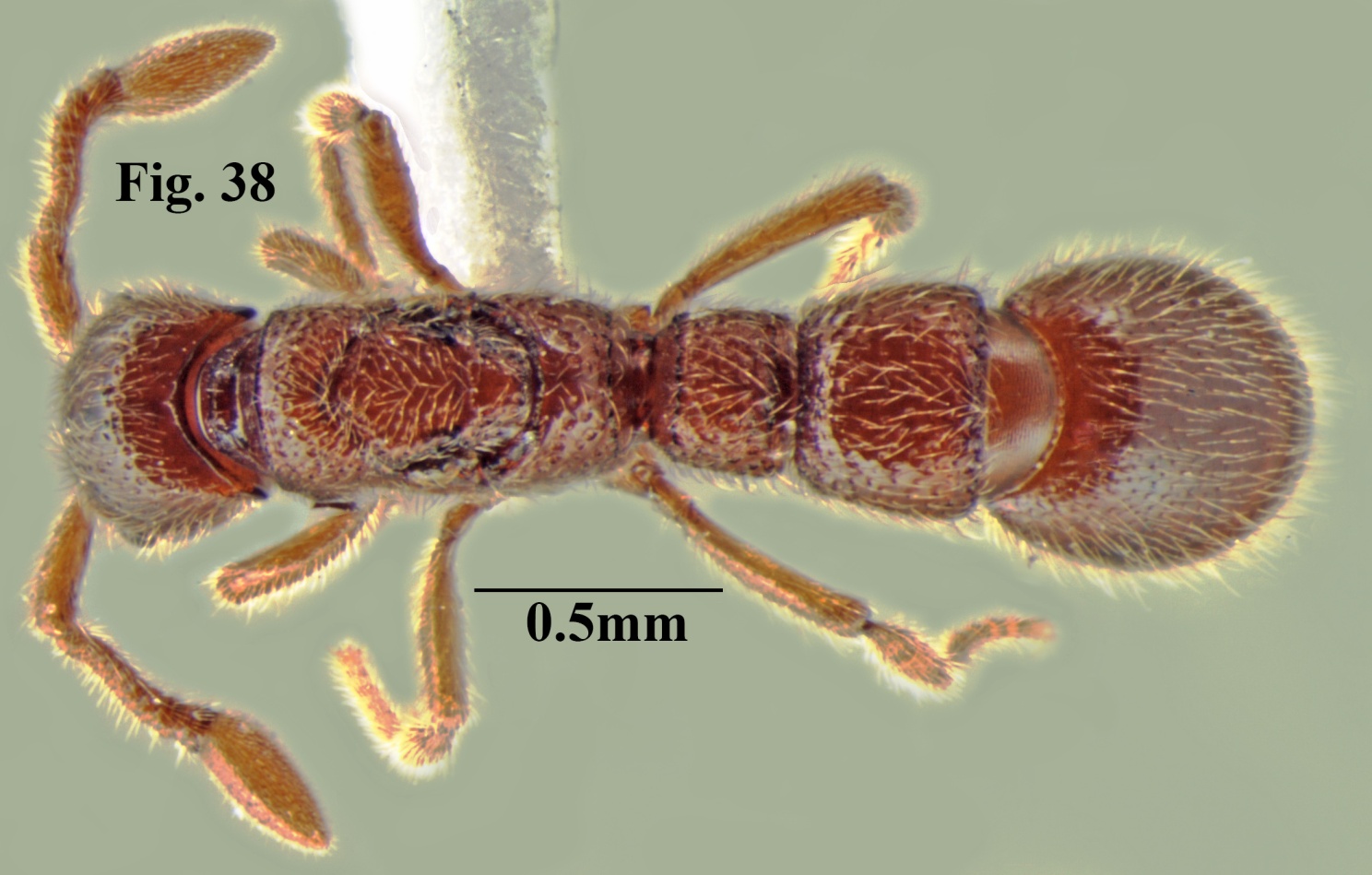


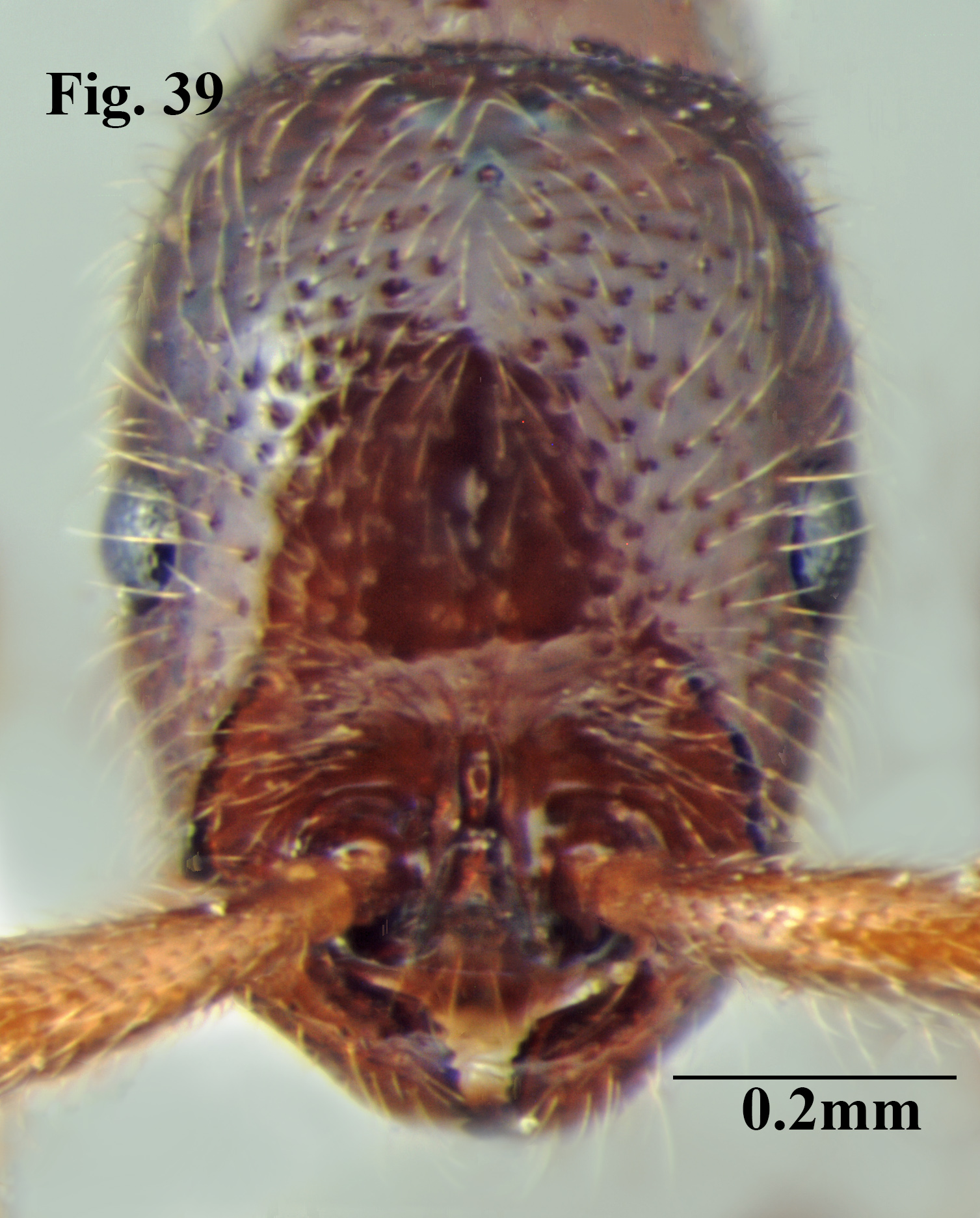


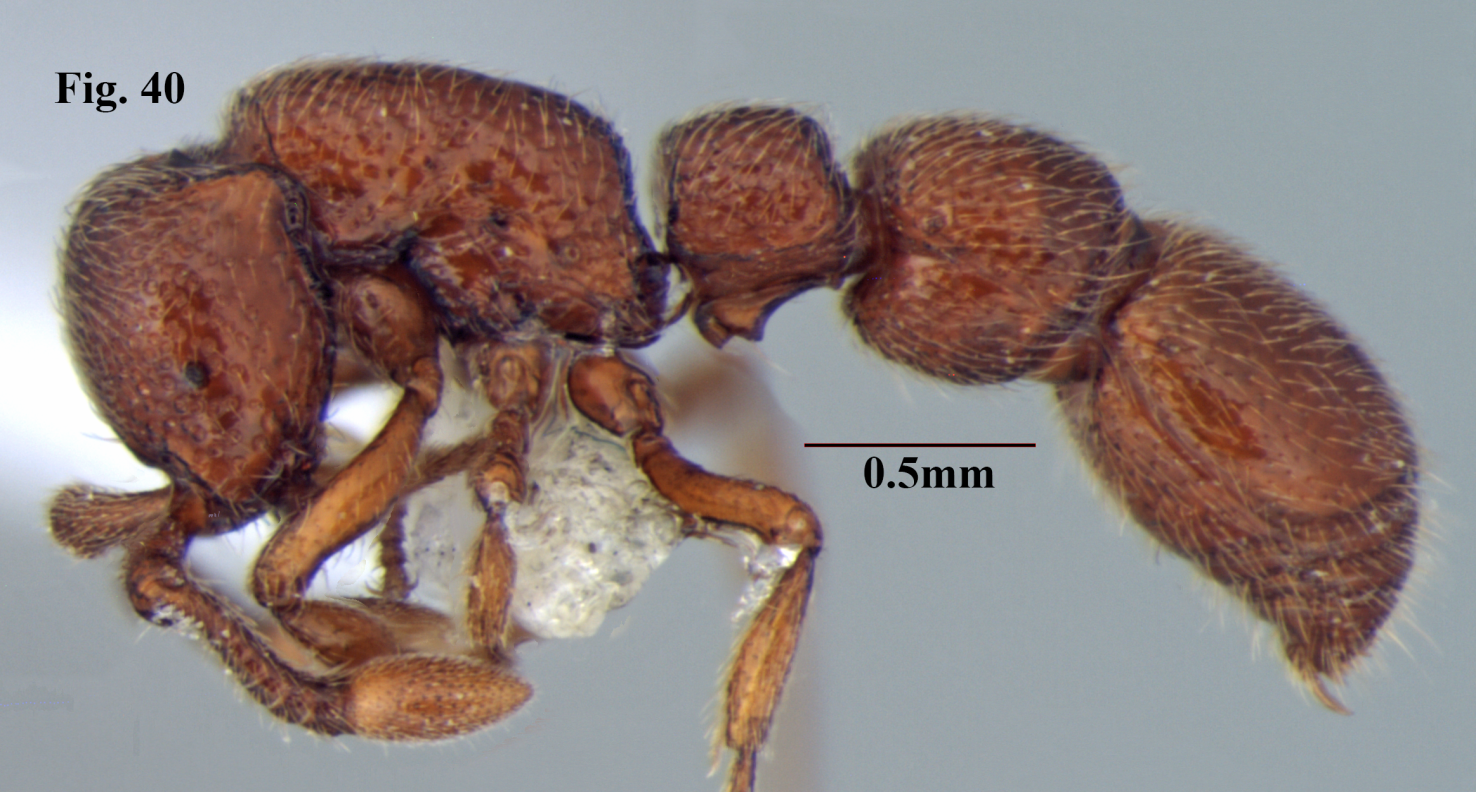


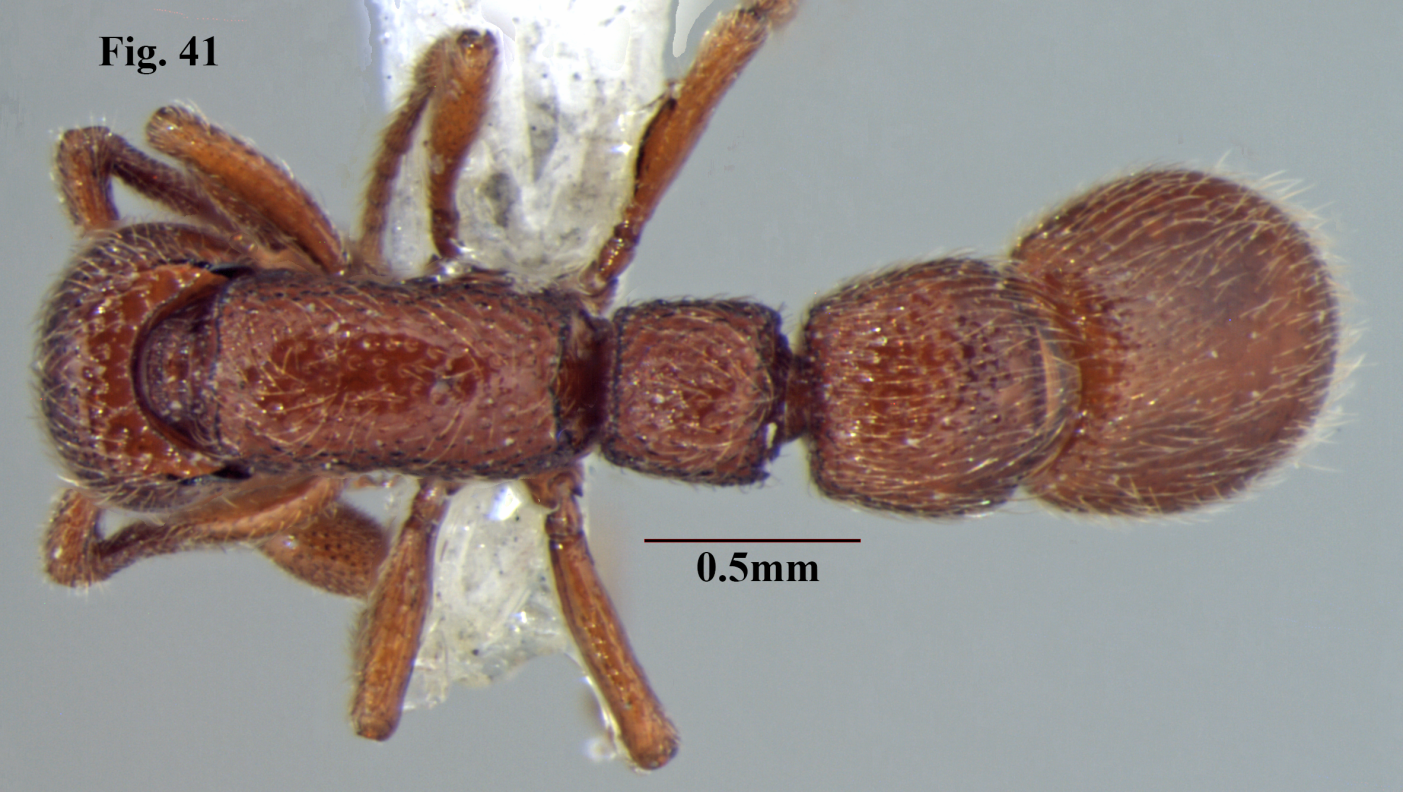


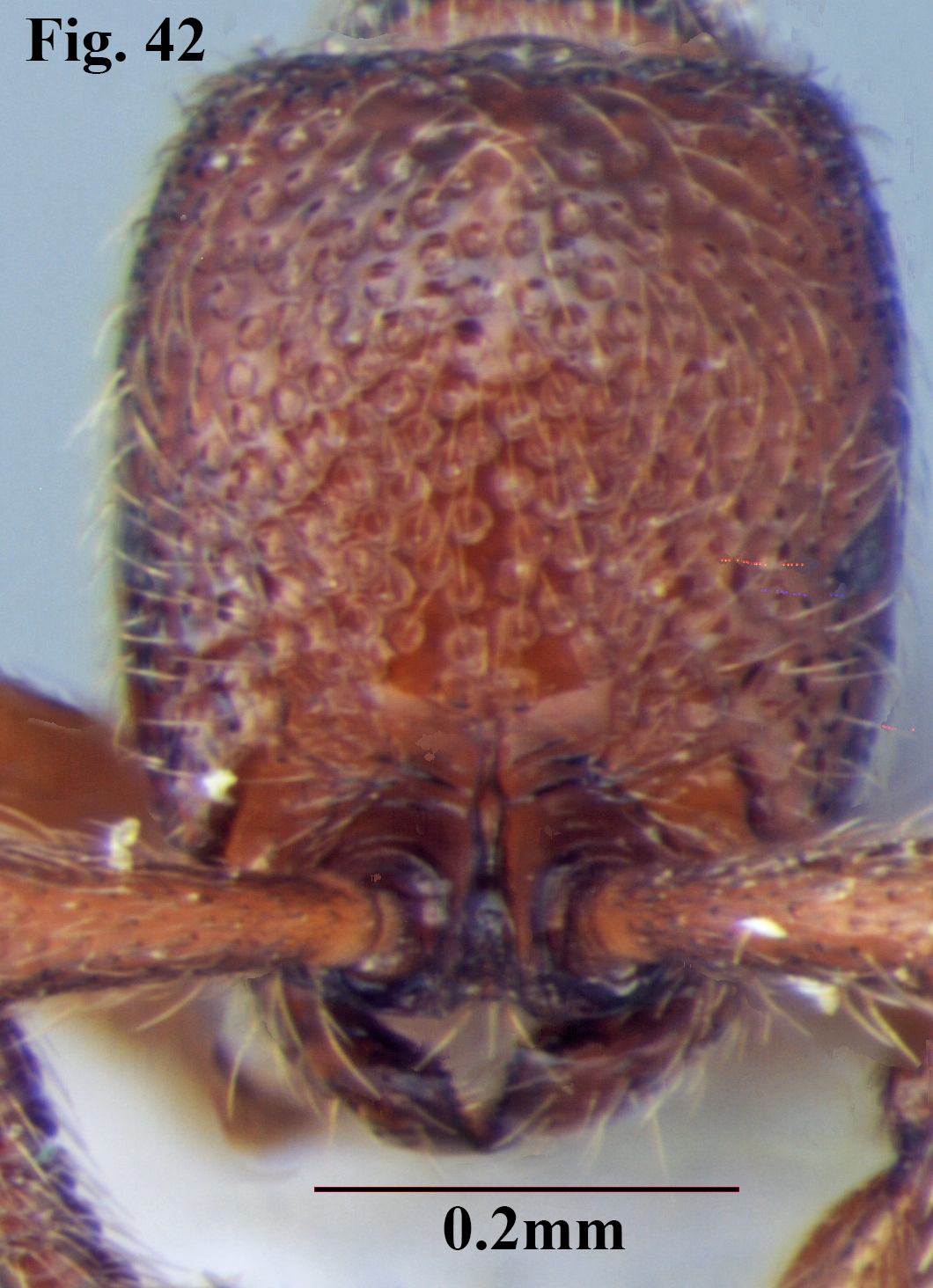

Supplement: Supplementary file 9 — Supplementary File 3 of image [file ZooKeys-336-079-s003.docx]

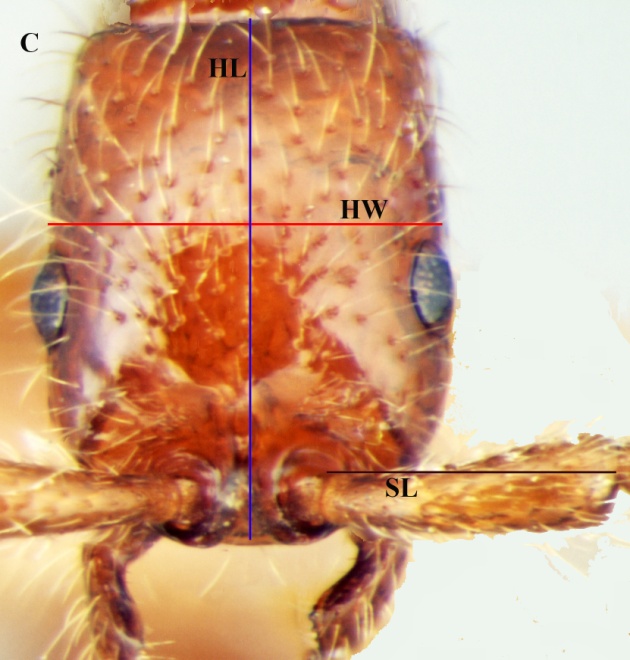


**
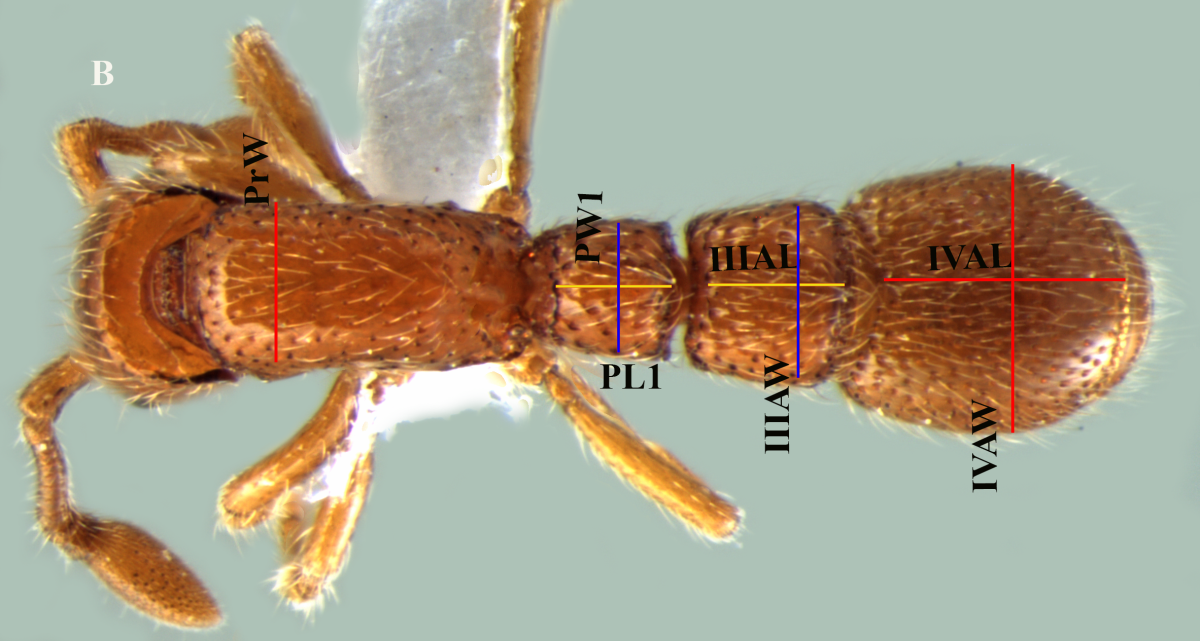
**

**
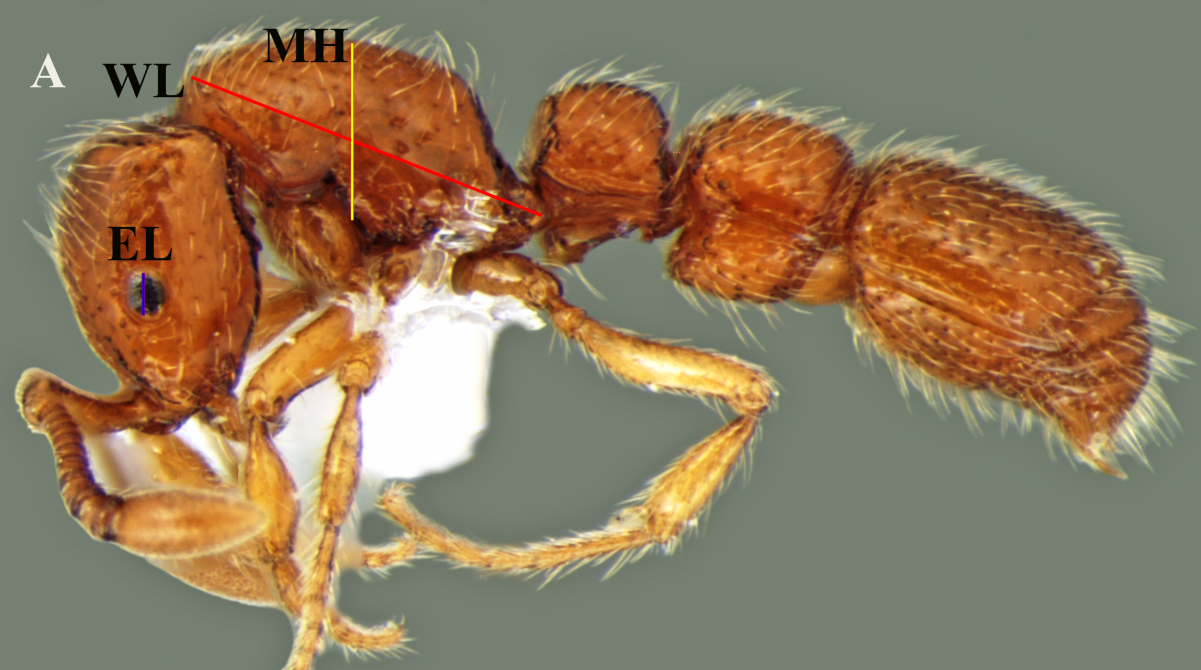
**


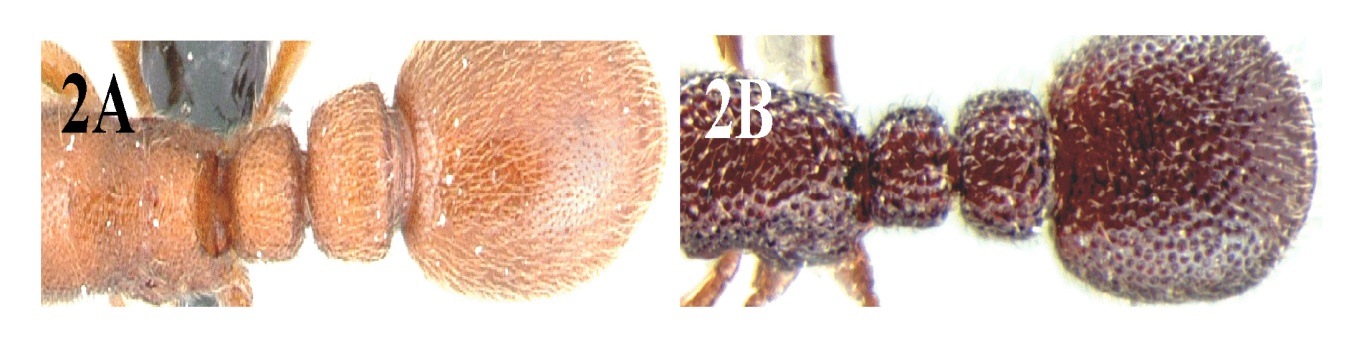


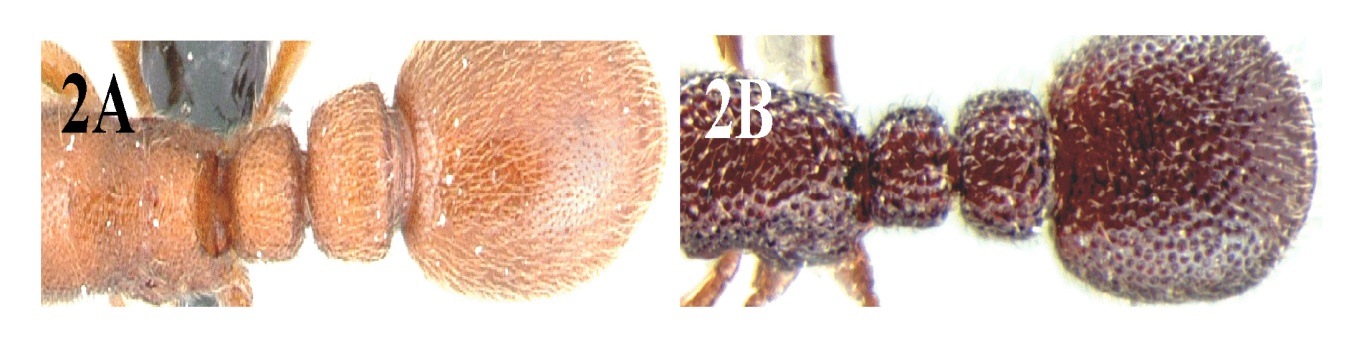


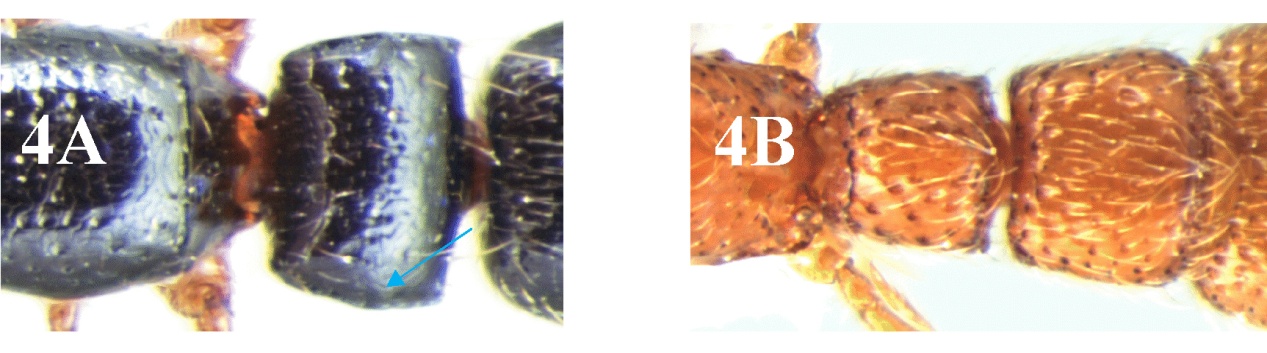


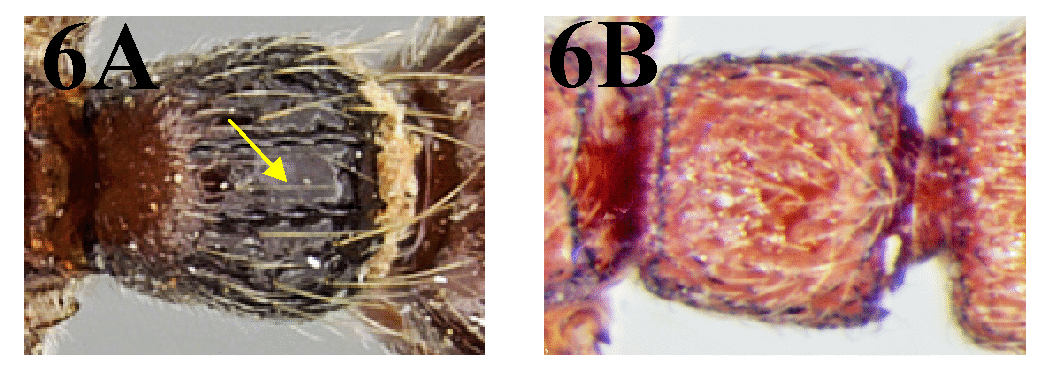


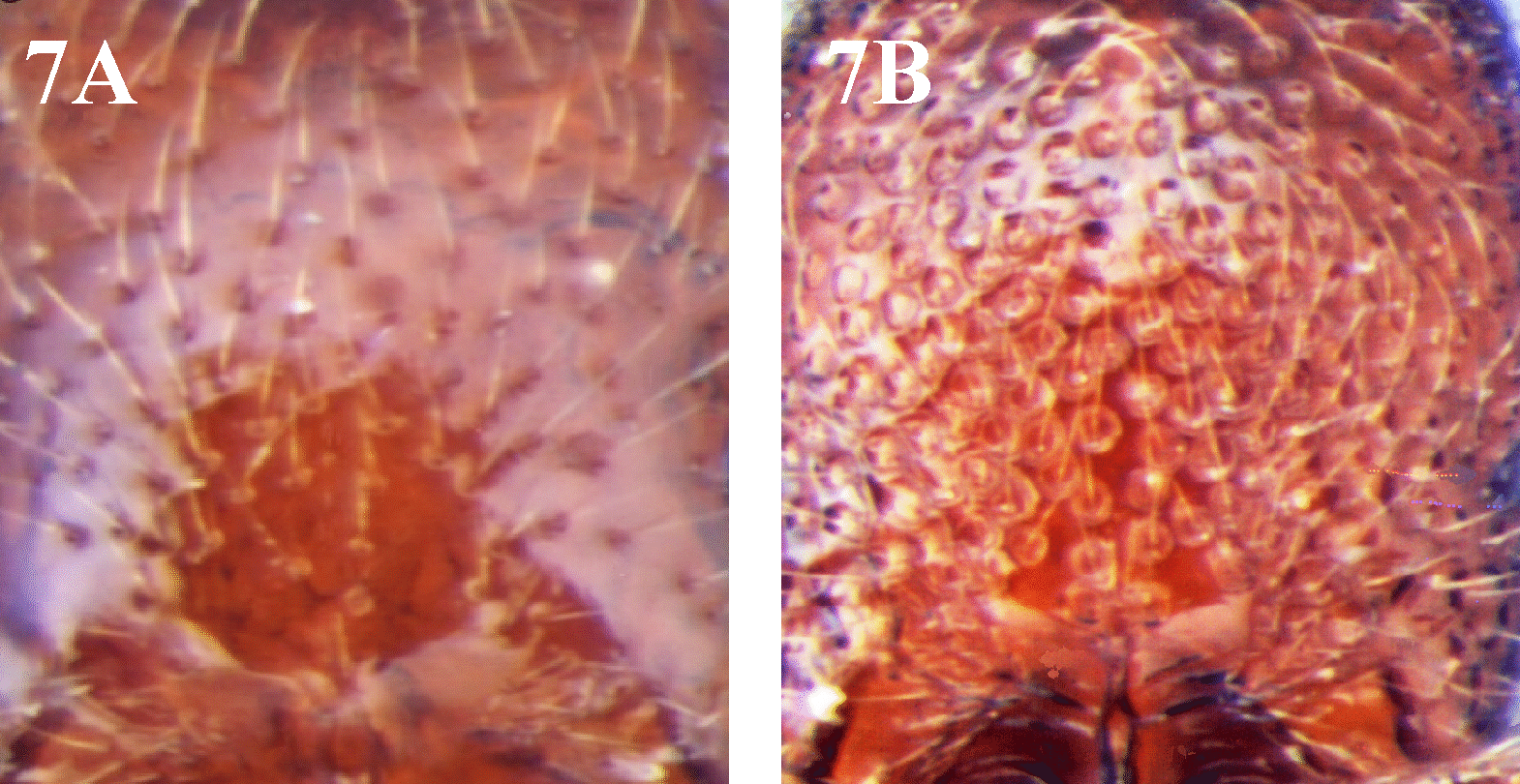


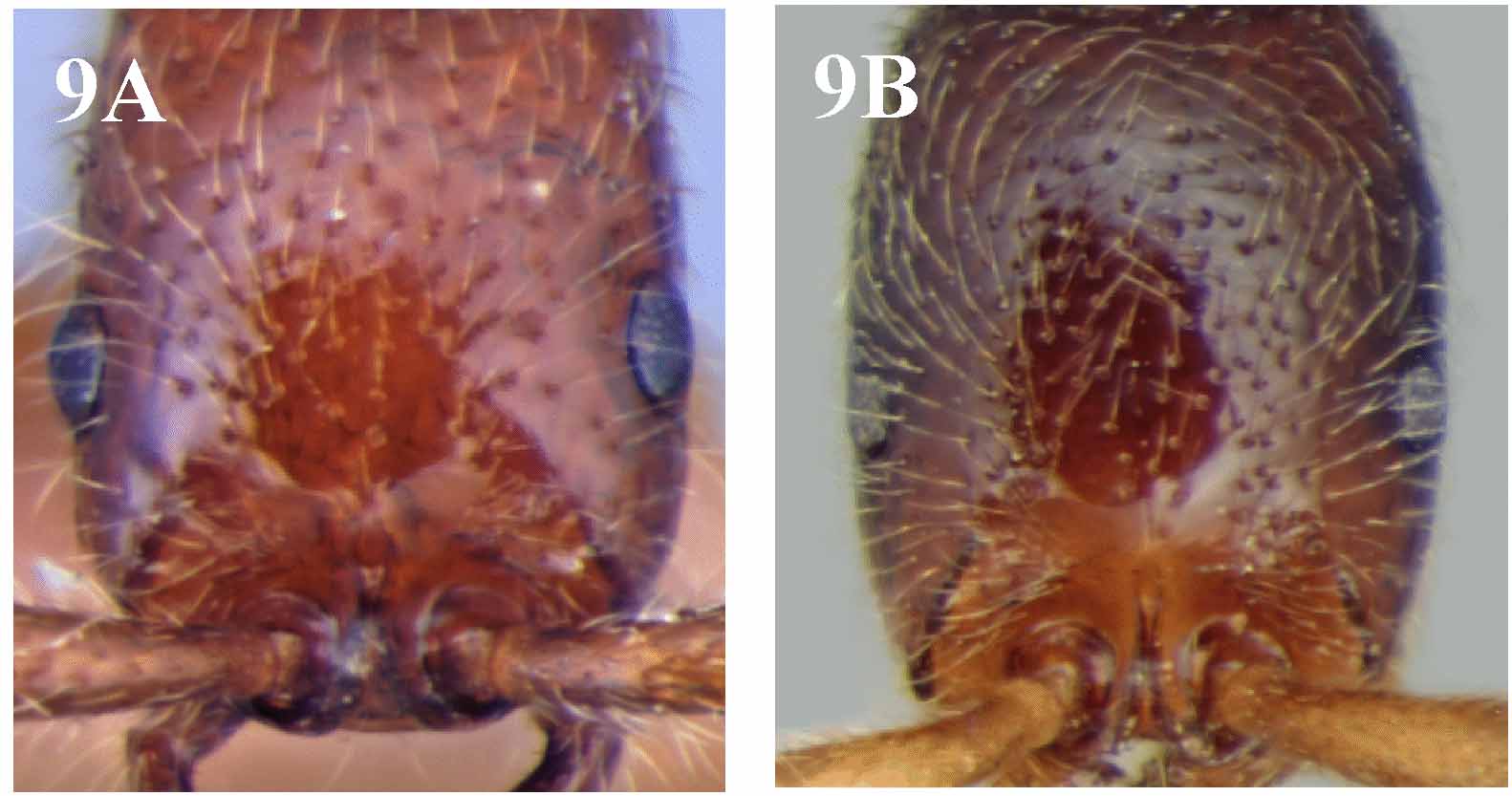


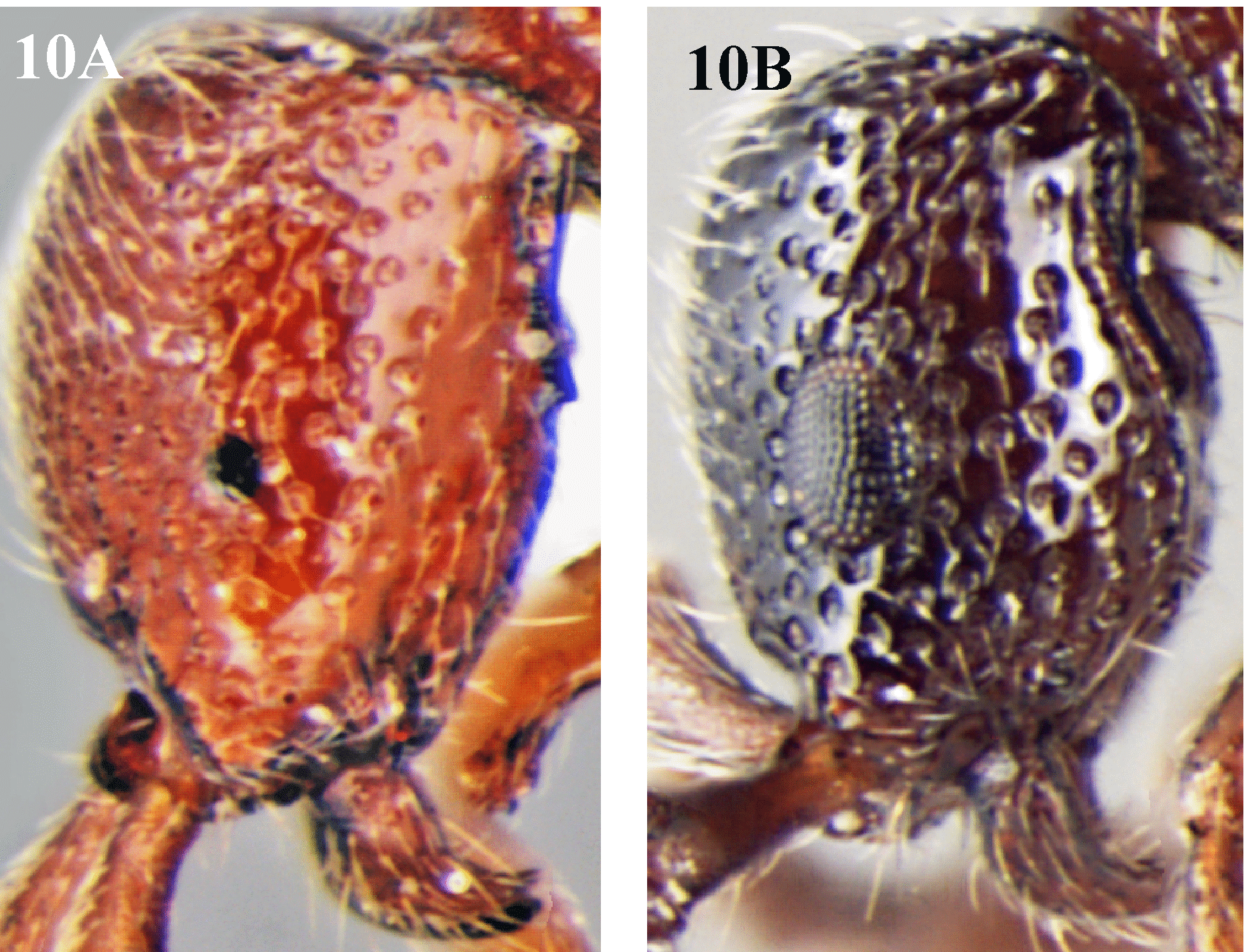


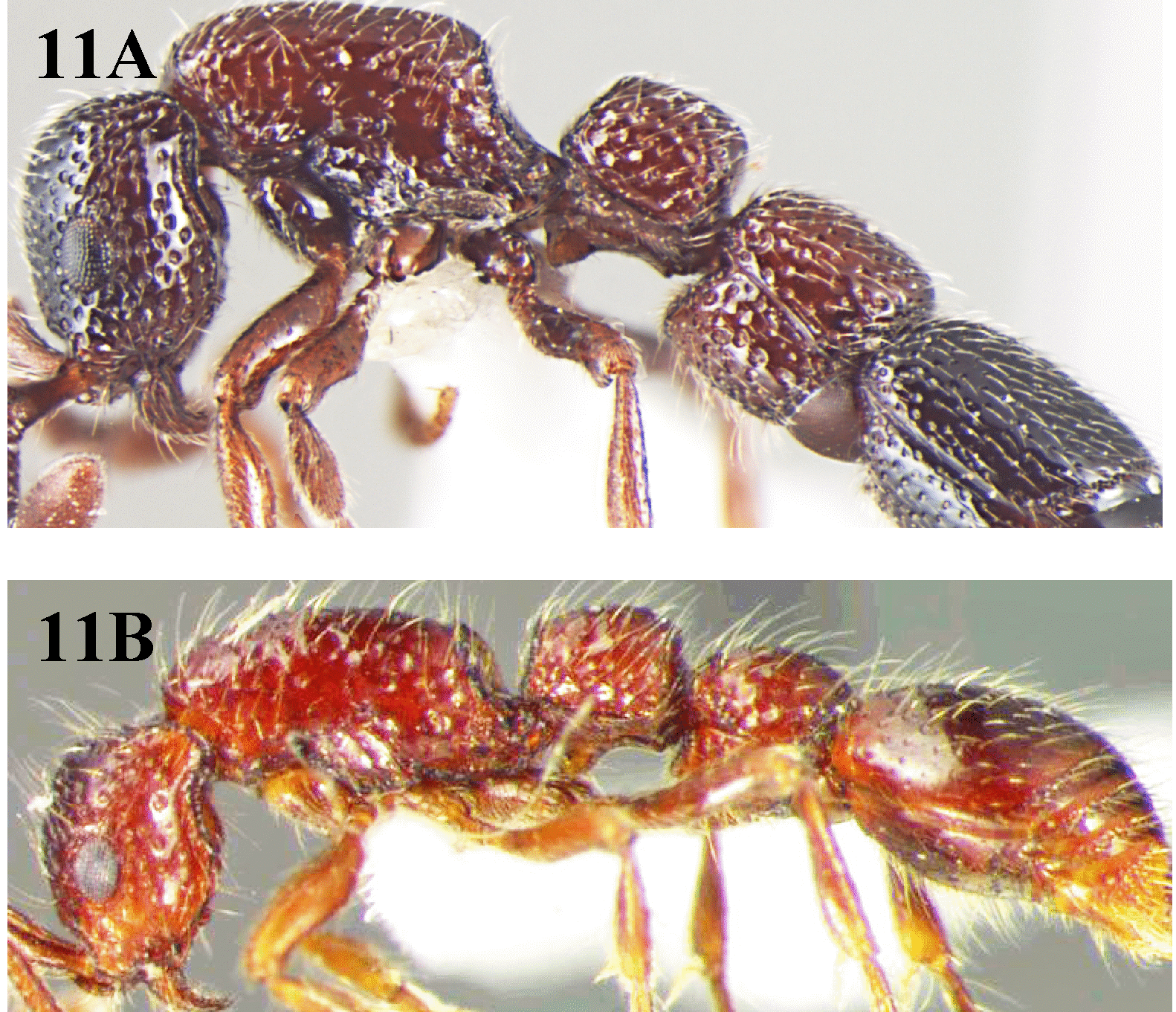


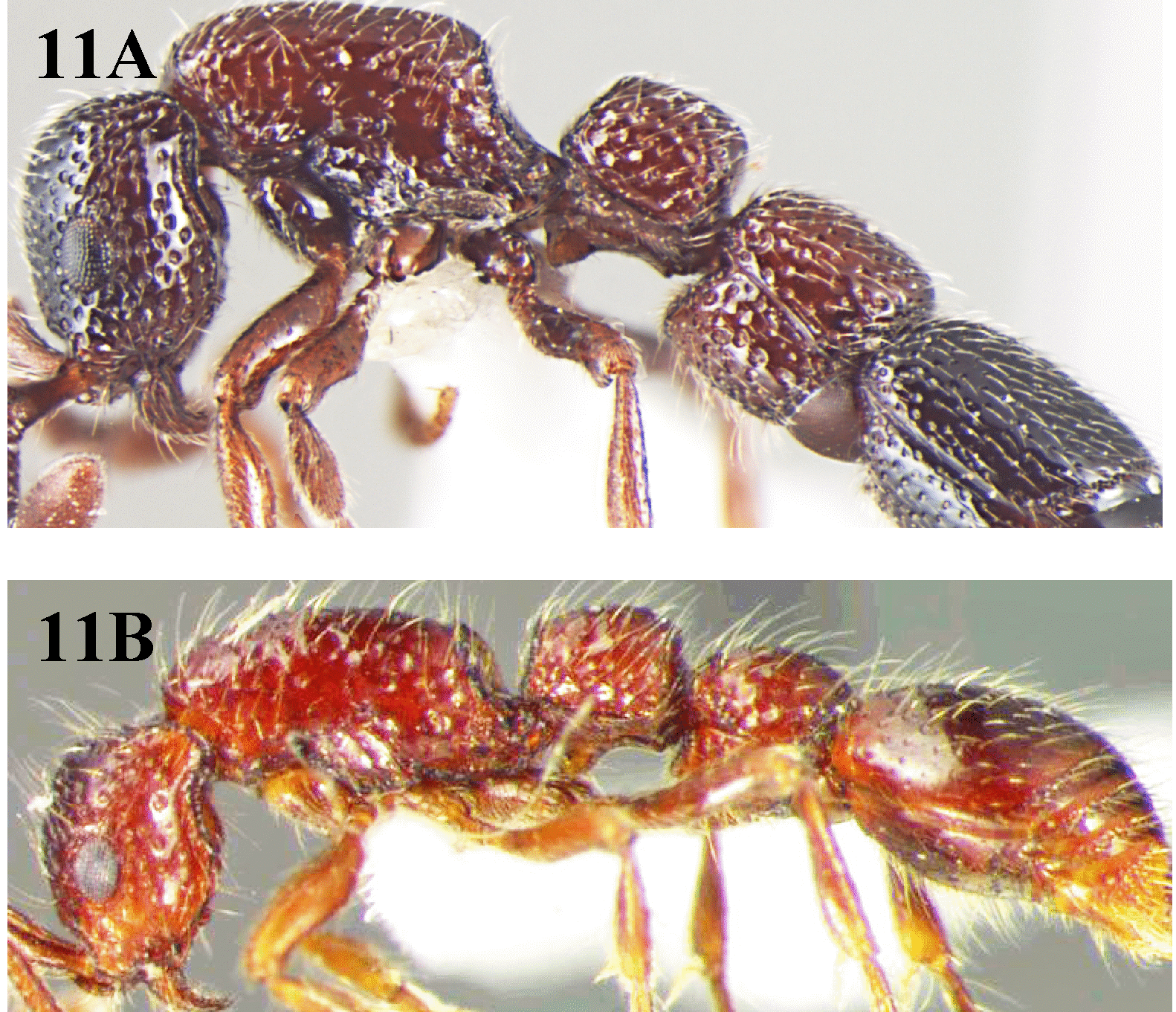

Supplement: Supplementary file 10 — Supplementary File of morphometrye [file ZooKeys-336-079-s004.docx]
