# Supplementary material for: Taxonomic studies on the ant genus Cerapachys Smith (Hymenoptera, Formicidae) from India
Source: Zookeys. 2013 Sep 27;(336):79–103. doi: 10.3897/zookeys.336.5719 (PMC3800781; doi:10.3897/zookeys.336.5719)
Supplement: Supplementary file 11 — Table-1 [file ZooKeys-336-079-s005.rtf]

S.no.	Species	Distribution	EQ	Q	M	W	
1	C. alii sp. n.	India, Kerala	-	-	-	+	
2	C. anokha sp. n.	India, Kerala	-	-	-	+	
3	C. aitkenii Forel, 1900	India, Kerala	-	-		+	
4	C. besucheti Brown, 1975	India, Tamil Nadu	+	-	-	+	
5	C. biroi Forel, 1907	Madagascar, Philippines, Puerto Rico, China, Nepal, India, Comoros, Seychelles, Mayotte, Japan, Viet Nam, Guam, Samoa, Marshall Islands, Northern Mariana Islands and Hawaii	+	+	+	+	
6	C. longitarsus (Mayr, 1879)	Philippines, New Guinea, Israel, Bangladesh, Thailand, Saudi Arabia, Egypt, United Arab Emirates	+	+	+	+	
7	C. nayana sp. n.	India, Kerala	+	-	-	+	
8	C. indicus Brown, 1975	India, Kerala	-	+	-	+	
9	C. schoedli sp. n.	India, Kerala	-	-	-	+	
10	C. seema sp. n.	India,Tamil Nadu	+	+	-	+	
11	C. sulcinodis Emery, 1889	Philippines, Indonesia China, India, Viet Nam and Thailand	+	+	+	+	
12	C. wighti sp. n.	India, Kerala	-	-	-	+	


Table I: Distribution of Cerapachys species. EQ= ergatoid queens; Q= queens; M=male; W=worker; + indicates reported and – indicates not reported.
