# Supplementary material for: Taxonomic studies on the ant genus Cerapachys Smith (Hymenoptera, Formicidae) from India
Source: Zookeys. 2013 Sep 27;(336):79–103. doi: 10.3897/zookeys.336.5719 (PMC3800781; doi:10.3897/zookeys.336.5719)
Supplement: Supplementary file 12 — Figure 43 [file ZooKeys-336-079-s006.rtf]

Figure 43.  Graph plotted on evaluating morphometric data shows less affinity of ergatoid queens with workers compared with gyne/queen. 	
